# Context-dependent protein structure prediction analysis and complex stoichiometry inference with MultimerMapper

**DOI:** 10.3389/fbinf.2026.1914731

**Published:** 2026-10-07

**Authors:** Elvio Rodríguez Araya, Esteban Serra

**Affiliations:** 1 Instituto de Biología Molecular y Celular de Rosario, Consejo Nacional de Investigaciones Científicas y Técnicas (CONICET), Rosario, Argentina; 2 Facultad de Ciencias Bioquímicas y Farmacéuticas, Universidad Nacional de Rosario (UNR), Rosario, Argentina

**Keywords:** AlphaFold, complex stoichiometry, conformational change, interaction dynamics, protein-protein interaction, MultimerMapper, structure prediction, trypanosomatids

## Abstract

Advances in artificial intelligence (AI) have transformed the field of structural biology, notably with the accuracy level reached by AlphaFold in the prediction of protein structures. While cloud implementations have broadened access to these methods, there is still a lack of non-AI tools to systematically interpret and analyze the resulting data, especially under varying modeling contexts. Here, we present MultimerMapper, a pipeline and software suite designed to generate, extract, integrate, analyze, and visualize the behavior of multimeric systems in ensembles of overlapping protein structure predictions. It utilizes statistics, graph theory, and novel algorithms to interpret how protein-protein interfaces and protein conformations behave across different modeling contexts, under the premise that they vary depending on the components introduced into the predictions. Starting from a list of input sequences, a stoichiometric space exploration algorithm guides users in the generation of highly informative structure prediction ensembles. Through interactive visualizations, MultimerMapper highlights higher-order subassemblies, infers stoichiometries, and identifies alternative interaction modes and conformational changes statistically associated with the presence or absence of specific protein partners. MultimerMapper offers a new perspective on the dynamic nature of predicted protein complex structures, supporting researchers in the exploration of functional mechanisms and assembly paths.

## 1 Introduction

AlphaFold2 (AF2) has increased protein structure prediction accuracy to unprecedented levels, providing the opportunity to obtain reliable structural information without the need for experimental determination (Jumper et al., 2021). AF2-multimer (AF2m) extended this capability to protein complexes and protein-protein interaction prediction (Evans et al., 2021; Bryant et al., 2022a). Later, the implementation on cloud computing platforms has democratized their use in the scientific community, enabling researchers to run these models using only protein sequences as input (Mirdita et al., 2022). Built on similar principles, other powerful models have emerged, including RoseTTAFold (RF), RoseTTAFold2 (RF2), and RoseTTAFoldNA (RFNA) (Baek et al., 2023; Baek et al., 2021; Baek, 2022). The latest release, AlphaFold3 (AF3), continues this progression by refining prediction accuracy and extending coverage to other molecules beyond polypeptides (Abramson et al., 2024). These advances aim toward greater versatility in modeling diverse atomic systems while improving computational efficiency and accuracy.

Despite these gains, there are still challenges that need to be addressed. For example, current AI models by themselves still fall short in capturing the dynamic nature of protein interactions and conformational diversity (Chen et al., 2024). Furthermore, predictions of small complexes that involve just a few models are easily tractable and interpretable, but systematic analysis and interpretation of structural ensembles for large unknown complexes remains difficult (Shor and Schneidman-Duhovny, 2024; Peng et al., 2024; Bryant et al., 2022b), particularly when the only information of a system is a list protein sequences. Complexity increases further when transient or dynamic interactions and conformational changes are considered, as these can induce proteins to associate or dissociate from their partners in a time and contextual-dependent manner (Acuner Ozbabacan et al., 2011; Tompa, 2016; Fuxreiter, 2024). Additionally, competitive binding scenarios, where different proteins interact with the same protein surface, create situations where the simultaneous interaction becomes physically impossible (Xie et al., 2015). These factors can lead to ambiguity in model interpretation, making it difficult to extract biologically relevant stoichiometries or interaction patterns. Even though some methodological approaches were developed (Chim and Elofsson, 2024; Liu et al., 2026), the prediction of stoichiometric coefficients when prior experimental data or templates are unavailable remains unsolved.

A relevant approach that inspired this work is the combinatorial assembly implemented by CombFold, which addresses some of these issues by assembling large complexes using overlapping subcomplex predictions (Shor and Schneidman-Duhovny, 2024). Even so, this method often requires some prior knowledge of complex stoichiometry, and ultimately generates static models without providing an explicit interpretation of interaction dynamics and conformational variability (Shor and Schneidman-Duhovny, 2024; Bryant et al., 2022b). We hypothesized that these overlapping structural datasets can also be used to explore how predicted interactions and conformations change depending on the modeling context, a process that we refer to as ‘context-dependent structure prediction analysis,’ which can be applied as a complementary approach to obtain meaningful biological information. Under this premise, we developed a method that systematically samples the stoichiometric space to capture and interpret these variations.

Before the initiation of this work, we intended to predict the structure of unknown trypanosomatid complexes, with the aim of designing wet-lab experiments (Rodríguez Araya et al., 2023). Soon, we faced the challenges already mentioned (large assembly complexity, transient, dynamic and competitive interactions; conformational changes and stoichiometric coefficient inference), inspiring the development of MultimerMapper, an approach to analyze structural variability in predicted protein complexes under different modeling conditions, with a particular focus on protein interfaces. We kept trypanosomatids as our training and validation background due to their significance as disease-causing organisms, our expertise in their complexes and their high evolutionary divergence from model organisms (da Silva et al., 2017). This divergence is reflected in the low protein sequence identity compared to model organisms, but conserving the structural features of core eukaryotic complexes (Rodríguez Araya et al., 2023), providing an excellent opportunity to validate our approach’s robustness. We further tested the method using non-trypanosomatid complexes, obtaining quantitative evidence that demonstrated its general applicability while delineating its strengths and areas for improvement. With this methodology, we attempt to address common limitations in the field, especially for highly dynamic complexes with unknown stoichiometries (Shor and Schneidman-Duhovny, 2024).

## 2 Materials and methods

### 2.1 Protein structure prediction

Structure predictions were performed with AlphaFold3 server (Abramson et al., 2024) and a local instance of AlphaFold2-multimer v3 (Evans et al., 2021). For AF2m, we used as input the multiple sequence alignments (MSAs) described by Bryant et al. (Bryant et al., 2022a), without using templates. For this, the Discoba database (Whee and ler, 2021) was modified to allow sequence matching and improve alignment coverage within the trypanosomatid taxonomic group. The MSA generated using the modified database was concatenated with the MSA generated by the ColabFold MSA server (Mirdita et al., 2022), as described by Wheeler et al. (Whee and ler, 2021). A full description of the MSA generation and the effects in AF2m metrics is available in the Supplementary Material. For each protein combination, 5 models were generated, using a maximum of 20 recycles, stopping recycling if two successive models reached a ΔRMSD < 0.5 Å. The runs were executed within Amazon Web Services (AWS) using g4dn.12xlarge, g5.48xlarge, p3.2xlarge, p3.16xlarge, or p3dn.24xlarge instances, depending on the dataset size and the total residues of individual predictions.

### 2.2 Models pairwise decomposition and metrics extraction

Each model of N protein chains ( $h_{1}$ , $h_{2}$ , …, $h_{N}$ ) was decomposed into all possible paired combinations. For each chain pair $h_{p},h_{q}$ , the combined atomic coordinates of $h_{p}$ and $h_{q}$ were extracted into a sub-model, along with their per-residue pLDDT values, and the two inter-chain off-diagonal submatrices of the model’s PAE matrix corresponding to residue pairs i,j of the chain $h_{p}\to h_{q}$ and $h_{q}\to h_{p}$ , respectively. One of the submatrices was transposed, and the minimum inter-chain PAE matrix was calculated by assigning the minimum observed value for each pair i,j . The overall minimum inter-chain PAE value (miPAE) was then calculated by taking the smallest element of the resulting matrix. The predicted docking coefficient (pDockQ) was also computed (Bryant et al., 2022a). In addition, the model’s pTM and ipTM values were extracted, ranking them based on the ipTM value relative to the other models within each prediction, assigning rank = 1 to the model with the highest ipTM. In the case of dimers (2-mers), models weren’t split into sub-models, as they represented pairwise interactions directly, and the same metrics described above were computed.

For each sub-model (or 2-mer), the identifier corresponding to each protein, the identifiers of each chain, the protein composition of the original model, the sub-model atomic coordinates, the minimum inter-chain sub-PAE matrix, rank, pTM, ipTM, miPAE, and pDockQ were stored in tabular format for further processing.

### 2.3 Protein-protein interaction and residue-residue contact prediction

PPI detection was performed for each pair of protein entities P,Q within each prediction composed of 5 models that had at least one chain of P and one of Q . By default, for 2-mers, the PPI between P and Q was considered positive in a prediction if at least 4 models had $miPAE\leq miPAE_{cutoff}$ (AF2m: $miPAE_{cutoff}=9.7\;Å$ for FPR≅0.05 and TPR≅0.61 at $N_{models}=4$ / AF3: $miPAE_{cutoff}=7.5\;Å$ for FPR≅0.05 and TPR≅0.61 at $N_{models}=4$ ). For N-mers, the PPI between P and Q was considered positive if at least 4 models had at least one decomposed pair of chains of P and Q that exceeded the established miPAE and $N_{models}$ cutoff values.

In cases where no PPI was detected for a pair within a prediction, all P,Q sub-models within the same prediction were considered to have no residue-residue contacts (RRCs). Otherwise, the center of mass per residue (centroid) of each chain in each decomposed model was calculated and an intercentroid distance matrix D of dimensions $L_{P}\times L_{Q}$ was constructed, where $L_{P}$ and $L_{Q}$ are the sequence lengths of P and Q , respectively. RRCs were considered to be those pairs of residues i,j with intercentroid distances $D_{ij}\leq 8\;Å$ and $miPAE_{ij}\leq miPAE_{cutoff}$ . The RRCs of each sub-model were represented in a contact matrix of dimensions $L_{P}\times L_{Q}$ , where 1 or 0 was assigned to each position i,j , depending on whether the residue pair was in contact or not, respectively.

### 2.4 Protein segmentation and domain detection

The segmentation of a protein P of sequence length $L_{P}$ into compact and well-structured segments separated by disordered segments was carried out by processing the intra-chain PAE matrix of its reference structure with a graph-based community clustering algorithm, modified from the algorithm described in https://github.com/tristanic/pae_to_domains. First, the reference structure was defined as the chain of P with the highest average pLDDT. Then, a graph with $L_{P}$ vertices (one per residue) was constructed and only the residue pairs i,j with $PAE_{ij}<5\;Å$ were connected with edges, assigning each pair a weight $w_{ij}=1/PAE_{ij}$ . Residues belonging to the same communities were identified using the Leiden clustering algorithm (Traag et al., 2019), controlling the granularity with a resolution parameter $g_{r}$ scaled in 1/100 (smaller $g_{r}$ → fewer and larger domains/larger $g_{r}$ → more and smaller domains). Finally, residue clusters from a small community (usually corresponding to disordered loops) surrounded by a larger community were integrated into the larger community.

To fine-tune domain assignment using the $g_{r}$ value, 2D visualizations were generated with the intrachain PAE matrix heat map and 3D visualizations representing the protein carbon backbone, both showing the assigned domains with different colors. This allowed interactively assigning the best $g_{r}$ values for each protein entity (Supplementary Figure 3).

### 2.5 RMSD pseudo-trajectories and bias in partners distribution (BPD)

For each protein entity P present in a set of c predictions with different combinations of n protein entities, RMSD pseudo-trajectories were constructed as follows. Atomic coordinates of all chains of P present in the c predictions were isolated, resulting in a list of m isolated models ( $P_{1},P_{2},\ldots ,P_{m}$ ), registering the stoichiometric coefficient of each protein entity present in the original combination. The reference structure ( $P_{ref}$ ) was identified as the model with the highest average pLDDT, the atomic coordinates of the α -carbons of each model $P_{i}$ were structurally aligned with those of $P_{ref}$ using the Kabsch algorithm (Kabsch, 1976), and the $RMSD_{i}$ value was calculated. The m models were sorted by ascending RMSD values, resulting in a pseudo-trajectory of m frames sorted by structural similarity. For each frame j , the per-residue pLDDT, the average pLDDT, and the radius of gyration (ROG) were recorded/computed. In addition, the Bias in Partner Distribution (BPD) was calculated for each frame j and each of the n protein entities. This parameter measures how locally enriched ( BPD≅1 ) or depleted ( BPD≅−1 ) a partner k is along the pseudo-trajectory with respect to its global frequency $f^{global}$ . To do this, an n×m boolean matrix was constructed, recording for each j -th frame whether the k -th protein entity was present or not in the original prediction. For each protein entity k , its $f^{global}$ was calculated as

$$f_{k}^{global}=\frac{1}{m}\sum_{j=1}^{m}presence\left(k,j\right)$$

where

$$presence\left(k,j\right)=\left\{\begin{array}{ll} 1, & \text{if }k\text{ is present in the original prediction of frame }j\\ 0, & \text{otherwise} \end{array}\right.$$

In the case where k=P , k was considered present only if k was found in at least two units. On the other hand, the local frequency ( $f^{local}$ ) was calculated in a rolling and centered manner for different window values $w\in \left\{5,10,15,20\right\}$ as

$$f_{k}^{local}\left(t\right)=\frac{1}{w}\sum_{j=t-h}^{t+h}presence\left(k,j\right)\;\text{for }t=\left\{h+1,h+2,\ldots ,m-h\right\}$$

where h=⌊w/2⌋ and t is the frame adjusted to the window size to avoid exceeding the range $j\in \left[1,m\right]$ . The BPD was defined for each frame t as

$${BPD}_{k}\left(t\right)=\left\{\begin{array}{c} \frac{f_{k}^{local}\left(t\right)-f_{k}^{global}}{1-f_{k}^{global}},f_{k}^{local}\left(t\right)\geq f_{k}^{global}\\ \frac{f_{k}^{local}\left(t\right)-f_{k}^{global}}{f_{k}^{global}-1},f_{k}^{local}\left(t\right)<f_{k}^{global} \end{array}\right.$$

With the collected information, each BPD_k_, RMSD, per-residue pLDDT (in the form of a heat map), the average pLDDT and the ROG were plotted as function of frame j , adding a small gaussian noise to the BPD to avoid overlapping of the curves. A final structural alignment step was applied to smooth out alignment fluctuations due to disordered loops. Only the α -carbons of residues with the highest median pLDDT (consistently well predicted) and lowest interquartile range (low variability) were used simultaneously (see Supplementary Material for a detailed description of how to identify these core residues based on pLDDT to perform the final alignment).

If more than one domain segment was assigned to P by the domain detection algorithm, the same algorithm was run for each segment, but restricted to the residues and atoms within that segment, assigning a new reference structure for each domain.

### 2.6 Multivalency detection

Let P and Q be two identical or distinct protein entities that establish PPI in at least one of all 2-mer or N-mer combination predictions, the engagement of multivalent interactions were determined using the fraction of multivalent chains (FMC) as follows. All decomposed models of P and Q coming from N-mers that contained 3 subunits of P and Q (1P2Q or 2P1Q) were taken, regardless of the presence or absence of other proteins. For each matching N-mer, the number of Q chains in contact with each P chain was counted, and *vice versa*. Two chains were considered in contact if they had at least 5 RRCs (see RRC prediction method). Multivalent chains ( $C_{2}$ ) were considered to be those in contact with ≥2 chains of the opposite protein entity, and monovalent chains ( $C_{1}$ ) were considered to be those in contact with ≤1 chain of the opposite protein entity. The FMC was defined as

$$FMC=\frac{m_{2}}{m_{1}{\;+\;m}_{2}}$$

where $m_{1}$ and $m_{2}$ are the number of observations for $C_{1}$ and $C_{2}$ , respectively. The P - Q pair was considered multivalent if FMC≥0.2 . In cases where FMC<0.2 and N-mers with more than 3 combined subunits of P and Q were available, it was recalculated using all N-mers and the P - Q pair was reconsidered using the same FMC cutoff.

### 2.7 Contact matrix clustering algorithm

Let P and Q be two protein entities, which can be identical or different, of sequence length $L_{P}$ and $L_{Q}$ , respectively, and interact with each other in a multivalent manner. Let M be the list of m Boolean contact matrices between P and Q ( $M=\left\{M_{1},M_{2},\ldots ,M_{m}\right\}$ ) generated with the RRC detection algorithm (see above) and T the list of atomic coordinates of the decomposed sub-structures of P and Q that gave rise to the matrices M ( $T=\left\{T_{1},T_{2},\ldots ,T_{m}\right\}$ ). The clustering algorithm described below was applied.

A distance matrix D between the m models was calculated from the RMSD values of the residues involved in contact in at least one of the matrices of each pair. To do this, for each pair of decomposed models u,v , the lists of residues involved in contacts ( Ω ) of P and Q were defined as

$$\Omega_{P}\left(u,v\right)=i\in \left\{1,\ldots ,L_{P}\right\}\;|\;\sum_{j=1}^{L_{Q}}M_{u}\left(i,j\right)+\sum_{j=1}^{L_{Q}}M_{v}\left(i,j\right)\geq 1$$

$$\Omega_{Q}\left(u,v\right)=j\in \left\{1,\ldots ,L_{Q}\right\}\;|\;\sum_{i=1}^{L_{P}}M_{u}\left(i,j\right)+\sum_{i=1}^{L_{P}}M_{v}\left(i,j\right)\geq 1$$

In other words, $\Omega_{P}$ and $\Omega_{Q}$ are the indices of residues i and j of P and Q , respectively, that are in contact in at least one of the two matrices. Atomic coordinates of the α -carbons of $\Omega_{P}$ and $\Omega_{Q}$ residues of each $T_{u},T_{v}$ pair were extracted, and the atomic coordinate lists ${xyz}_{u}\left(u,v\right)$ and ${xyz}_{v}\left(u,v\right)$ were constructed. Both lists have the same length for each u,v pair and are sorted in ascending order by index in Ω , concatenating first those residues of $\Omega_{P}$ and then those of $\Omega_{Q}$ , maintaining an element-by-element correspondence. The ${xyz}_{v}\left(u,v\right)$ coordinates were aligned to those of ${xyz}_{u}\left(u,v\right)$ using the Kabsch algorithm (Kabsch, 1976), and the RMSD was calculated. The process was repeated for each u,v pair, and the m×m -dimensional distance matrix D was constructed from the results.

D was used as input distance matrix for the bottom-up hierarchical clustering algorithm using the average linkage method. The number of clusters to explore was set between $K_{\min}$ and $K_{\max}$ , which depend on the maximum valency ( ψ ) of the chains observed during the multivalency detection algorithm (see above) and the number of matrices to be clustered ( m ), as follows:

$$K_{\min}=\max\left(2,\psi -2\right),K_{\max}=\min\left(m,\psi +3\;\right)$$

For each cluster number K , the average silhouette coefficient ( $\bar{s}$ ) was calculated from the resulting groups and the distance matrix, choosing the optimal number of clusters ( $K_{op}$ ) as the one that maximizes $\bar{s}$ .

Finally, the contact matrices for each cluster K were averaged to create the average contact matrices ( $\bar{M}$ ), which represent relative frequency of contacts by position i,j . That is, the one calculated as

$${\bar{M}}_{K}\left(i,j\right)=\frac{1}{m_{K}}\sum_{u=1}^{m_{K}}M_{u}\left(i,j\right)$$

where $m_{K}$ is the number of matrices in cluster K . A representative structure was taken for each cluster by taking the model whose contact matrix had the smallest Jaccard distance (intersection over union) to the binarized average matrix.

To construct the multidimensional scaling (MDS) visualizations, D was passed as argument of the MDS function of sklearn package, setting 2 as number of components. The resulting coordinate components of each matrix were plotted in a dispersion plot, coloring them by cluster.

### 2.8 Stability, convergence and stoichiometry inference

#### 2.8.1 N-mer stability definition

An N-mer composed of N protein chains ( $h_{1},h_{2},\ldots ,h_{N}$ ) with five available structural models was defined as stable if enough models produced fully connected inter-chain contact graphs independently. The definition is described below.

Each model of rank r (ipTM rank) was converted into an undirected graph ( $G_{r}$ ), representing the chains as nodes and connecting with edges those $h_{p},h_{q}$ pairs that had a number of contacts C between all their interchain residue pairs i,j that exceeded a cutoff value ( $C_{cutoff}=5$ by default). That is, nodes $h_{p}$ and $h_{q}$ were connected when

$$C_{ab}=\sum_{i=1}^{L_{a}}\sum_{j=1}^{L_{b}}C_{ij}\geq C_{cutoff}$$

where $L_{p}$ and $L_{q}$ are the sequence lengths of $h_{p}$ and $h_{q}$ , respectively; and $C_{ij}$ is given by

$$C_{ij}=\left\{\begin{array}{l} 1,\text{if }PAE_{ij}<PAE_{cutoff}\wedge d_{ij}<8\;Å\\ 0,\text{otherwise} \end{array}\right.$$

where $PAE_{ij}$ and $d_{ij}$ are the inter-chain PAE and inter-centroid distance values, respectively, for the residue pair i,j ; and $PAE_{cutoff}$ is the PAE cutoff value ( $PAE_{cutoff}=7.5\;Å$ by default). $G_{r}$ was considered fully connected if, for every possible pair of nodes, there is a path between them. Next, the number of $G_{r}$ fully connected ( R ) was quantified, considering the N-mer stable if $R\geq r_{cutoff}$ ( $r_{cutoff}=4$ by default). Otherwise, it was defined as unstable.

If N=2 (2-mers), the combination was considered stable if it exceeded the PPI detection cutoff (see PPI detection method).

#### 2.8.2 Stoichiometric space exploration algorithm and system convergence

Each system of n protein entities was analyzed using a pipeline consisting of rounds of protein structure prediction, analysis (PPI prediction, RRC prediction, and detection of stable N-mers), and generation of combinations (suggested N-mers) of the n entities based on observations during the analysis section. At the start of each new round, the suggested predictions were predicted, and then added to those from previous rounds into a new analysis stage.

Systems were initialized by predicting all possible pairwise combinations of the n entities (2-mers), including homodimers. By default, after detecting interacting 2-mers (see PPI prediction method), n suggestions of size N=3 (3-mers) were generated for each stable 2-mer by adding one unit of each possible protein entity. The system was represented as a directed graph, where the resulting stoichiometries (suggested combinations) were considered children of the stable 2-mers that gave them origin. In this graph, each daughter could have more than one parent, and unstable 2-mers did not generate daughter stoichiometries.

The suggestions were predicted and incorporated into a new round of analysis with the 2-mers. In this new round, stable 3-mers were detected and n new suggestions of N=4 were generated for each stable 3-mer, without generating children for unstable 3-mers. The process was repeated, increasing N+1 in each iteration and extending the graph with new daughter stoichiometries. The process stopped when the system reached convergence, that is, when no suggested stoichiometries remained unpredicted. Convergent stoichiometries of the system were identified as those stable stoichiometries where all of their children were unstable and none of their possible children were left unexplored.

For systems where all N=3 suggestions were unstable (e.g., nucleosome), in addition to the N+1 increments, N+2 increments were incorporated for each iteration. To do this, d extra suggestions were generated for each stable 2/N-mer by adding the stoichiometry of each possible stable 2-mer to the stoichiometry of the base stable 2/N-mer, where d is the number of stable 2-mers. N+2 suggestions were considered children of the N-level stoichiometries that gave rise to them only if no stable intermediate ( N+1 ) stoichiometry that could have given rise to them were found.

### 2.9 Symmetry fallback detection

Let P be a homooligomerizing protein entity, with Z−1 successive homooligomeric predictions of P available, ranging from the dimer (homo-2-mer) to the homooligomer of size Z (homo-Z-mer). The presence of a symmetry fallback was defined by detecting a drop greater than 20% in the estimated radius of the top-ranked model (ipTM rank) in any homo-N-mer ( $R_{N}$ ) relative to the estimated radius of its predecessor top-ranked model ( $R_{N-1}$ ). To estimate the radius, for each chain h of each rank 1 model, the center of mass (CM) of the domains involved in homooligomerization ( D ) was computed according to

$$CM_{h}=\frac{1}{m_{D}}\sum_{a\in D\in h}^{m_{D}}{xyz}_{a}$$

where the sum is performed over the atoms ( a ) of D of chain h , $m_{D}$ being the number of atoms in D , and xyz being their atomic coordinates. The global center of mass of the model was then calculated, restricting it to the domains involved in homooligomerization as

$$M_{global}=\frac{1}{{N\times m}_{D}}\sum_{a\in D}^{N\times m_{D}}{xyz}_{a}$$

The Euclidean distance ( d ) of each chain to the $CM_{global}$ was calculated, estimating $R_{N}$ with the average of the Euclidean distances ( $\bar{d}$ ):

$$d_{h}={\left\|CM_{global}-CM_{h}\right\|}_{2}\;R_{N}\equiv {\bar{d}}_{N}=\frac{1}{N}\sum_{h=1}^{N}d_{h}$$

In cases where a symmetry fallback was detected for an N-mer ( $R_{N}<0.8\times R_{N-1}$ ), it was determined which stoichiometry it resembled by comparing its radius ( $R_{F}$ ) with that of N-mers predecessors ( $R_{N<F}$ ). For this purpose, a 95% Student’s T confidence interval (CI) with N−1 degrees of freedom of $R_{F}$ was constructed and it was checked whether any $R_{N<F}$ was contained by CI. The first homooligomer (in decreasing N size order) of size T whose $R_{T}$ was contained by CI was assigned as the falling back stoichiometry, assigning the nomenclature $F_{f}T$ ( F falls back to T ) to indicate that the homooligomer of size F undergoes a symmetry fallback that makes it similar to the homooligomer of size T . If no $R_{N<F}$ was contained by CI, the radii were reestimated using a two-dimensional projection (see Supplementary Material and Supplementary Figure 1) and the process was repeated using the CI of the projected radius and the projected radii. In case neither of the two methods covers the R of a smaller homo-N-mer, the $R_{T}$ that minimizes the difference $\left|R_{F}-R_{N<F}\right|$ was chosen.

### 2.10 Correlation between PPI dynamics and protein presence using phi coefficient

The tendency to stabilize or destabilize a PPI between a pair of protein entities P and Q induced by the presence of a third protein entity S was quantified using the phi correlation coefficient ( φ ). To do this, all decomposed models of P and Q derived from N-mers were analyzed, and a 2×2 matrix was constructed representing the count of detection (Jumper et al., 2021) or non-detection (0) of PPIs in the columns, and the count of presence (Jumper et al., 2021) or absence (0) of S in the model in the rows. Then, φ was computed as

$$\varphi =\frac{n_{11}n_{00}-n_{10}n_{01}}{\sqrt{n_{1}\cdot {n\cdot }_{1}n_{0}\cdot {n\cdot }_{0}}}$$

where $n_{11}$ is the number of decomposed models in which a PPI was detected and S was present; $n_{00}$ is the number of those in whom PPI was not detected and S was not present; $n_{10}$ is the number of those in whom PPI was detected and S was not present; $n_{01}$ is the number in whom PPI was not detected and S was present; and $n_{1}\cdot$ , ${n\cdot }_{1}$ , $n_{0}\cdot$ , and $n_{\cdot 0}$ are the marginal totals.

In cases where P≠Q and $S\in \left\{P,Q\right\}$ , S was considered present in the model only if it was found in ≥2 units. For cases where P=Q=S , S was considered present in the model only if it was found in ≥3 units. PPI detection was defined as the presence of at least 5 residue-residue contacts between P and Q . The null hypothesis was no association between the presence of S and PPI detection ( $H_{0}:\;\varphi =0$ ), and the alternative hypothesis was association ( $H_{1}:\;\varphi \neq 0$ ), which was evaluated using the Chi-Square ( $\chi^{2}$ ) test. Values of φ>0 (p-val < 0.05) were associated with a positive correlation between the presence of S and the establishment of PPI, while values of φ<0 (p-val < 0.05) were considered a negative association.

### 2.11 Correlation between RMSD and protein presence using point-biserial correlation

The correlation between the conformational state of an ordered domain D in a protein entity P and the presence of another protein entity S was quantified using the point-biserial correlation coefficient ( $r_{pb}$ ). To do this, the segment corresponding to D was isolated from all m isolated chains of P , and the root mean square deviation (RMSD) value was calculated against the segment D of the reference structure. An m×2 table was then constructed with the RMSD values in one column and the presence (Jumper et al., 2021) or absence (0) of S in the model from which the isolated chain originated in the other. The coefficient $r_{pb}$ was computed as

$$r_{pb}=\frac{{\bar{RMSD}}_{1}-{\bar{RMSD}}_{0}}{s_{m}}\sqrt{\frac{m_{1}m_{0}}{m\left(m-1\right)}}$$

where $s_{m}$ is the standard deviation of all chains; ${\bar{RMSD}}_{1}$ and ${\bar{RMSD}}_{0}$ are the average RMSDs of the chains where S was present or absent, respectively; and $m_{1}$ and $m_{0}$ are the number of chains where S was present or absent, respectively.

Ordered domains of P were defined as those segments defined by the domain detection algorithm (see protein segmentation method) with $mean\left(pLDDT\right)>60$ values in the reference structure. Only the α -carbons of residues with pLDDT>70 were used to calculate the RMSD. In cases where P=S , S was considered present in the model only if found in ≥2 units. The null hypothesis was the absence of an association between the RMSD value and the presence of S ( $H_{0}:\;r_{pb}=0$ ), and the alternative hypothesis was the association ( $H_{1}:\;r_{pb}\neq 0$ ), which was tested using a T-test with m−2 degrees of freedom. Values of $r_{pb}>0$ (p-val < 0.05) were interpreted as a positive association (the presence of S is associated with RMSD values further from the reference) and values of $r_{pb}<0$ (p-val < 0.05) as a negative association (RMSD values closer to the reference).

### 2.12 Pairwise confidence profiles

Let P and Q be two protein entities, which may be identical or distinct, that were detected as interactors using the PPI detection method (see PPI prediction method), each present in at least one subunit in c structural predictions. Each structural prediction consists of five models and was generated using different combinations of n protein entities. The construction of confidence profiles for the P - Q pair is described below.

The pLDDT values per residue were extracted from all P and Q chains, and the resulting values were grouped according to the structural prediction (combination of proteins) and model number. For each group, the average pLDDT, its standard deviation (SD), median, first quartile (Q1), and third quartile (Q3) were calculated.

From the PAE matrix of each model, the two PAE submatrices corresponding to the interchain residues of each pair of P and Q chains were extracted. The two matrices were combined by transposing one of them and maintaining the minimum value for each residue pair. The average PAE value of each resulting matrix (aiPAE) was then calculated, and the miPAE values were extracted. The aiPAE and miPAE values were grouped by structural prediction and model, and the mean, SD, median, Q1, and Q3 were calculated for each group.

The pLDDT, aiPAE, and miPAE profiles were constructed by plotting the average pLDDT and its SD (or median and interquartile range) as a function of model number and colored according to the protein combination of each structural prediction.

### 2.13 Default parameters

Default parameter values for all cutoffs and configurations described in this work are summarized in Table 1.

**TABLE 1:** Default parameters and cutoffs used by MultimerMapper.

Manuscript section	Parameter	Description	Configuration variable	Default value
2.4.3/1.3/1.8.1	N_models_/r_cutoff_	Min. number of models (of 5) that must exceed the interaction cutoff to call a PPI	N_models_cutoff	4
2.4.3/1.3	miPAE_cutoff_	Minimum interaction PAE cutoff at FPR = 0.05 by default	PAE_cutoff	7.5 Å (AF3)
2.7.1/1.8.1	Contacts number cutoff (*C*_*cutoff*_)	Min. residue-residue contacts between two chains to connect them in the stability graph	Nmers_contacts_cutoff	5
2.7.2/1.9	Symmetry Fallback Threshold	Drop (fraction) in estimated radius required to consider a symmetry fallback	SF_drop_threshold	0.2
2.6.1/1.3	Contact distance cutoff	Max. inter-centroid distance to call a residue-residue contact	contact_distance_cutoff	8.0 Å
2.6.1/1.3	Contact PAE cutoff	Max. inter-chain PAE to call a residue-residue contact	contact_PAE_cutoff	7.5 Å (AF3)
2.6.2/1.6	FMC threshold	Min. FMC to classify a pair as multivalent	multivalency_metric_threshold	0.2
2.5.1/1.4	Resolution ( $g_{r}$ )	Leiden clustering resolution for PAE-based domain segmentation	graph_resolution	0.075
2.7.1/1.8	Discard non-interacting proteins	If true: excludes proteins with no PPI evidence at all from the combinatorial space	combine_only_detected_ppi_proteins	False
2.7.1/1.8	Use fully connected combinations	If passed: discards combinations that mix proteins from separate PPI sub-networks (CLI flag)	--use_fully_connected_combinations	False
2.7.1/1.8	Combination order	Max. increment size added per iteration to stable stoichiometries (+1, +1 and +2, etc.)	max_combination_order	1
2.8.3/1.11	Domain pLDDT cutoff	Minimum average pLDDT to consider a protein segment to compute r_pb_ coefficient	domain_RMSD_plddt_cutoff	60
2.8.3/1.11	Disordered segment pLDDT cutoff	Minimum pLDDT to consider a residue to be used to compute the r_pb_ coefficient	trimming_RMSD_plddt_cutoff	70
3	φ-coefficient computation method	Controls if the phi-coefficient and p-value are computed per model (True) or per prediction (False)	use_phi_per_model	True

Parameter: Name of the parameter. Description: Brief description of the parameter. Configuration variable: name of the variable located in the default_settings.py file from the source code or in the command-line interface (CLI). Default value: default value assigned to the variable. Manuscript section: section of the manuscript where the parameter is described.

## 3 Results

### 3.1 Context-dependent protein structure prediction

To understand the principles of our approach, we will define some ideas. MultimerMapper introduces the concept of “predicted dynamic interactions”, based on the hypothesis that protein-protein interactions (PPIs) can engage or disengage depending on the context in which they are modeled. The basis is to compare pairwise interactions across different prediction scenarios, *e.g.*, with or without a third protein partner. This allows the extraction of valuable information about conformational dynamics, complex assembly dynamics and stoichiometry, minimizing the reliance on prior knowledge about the system.

#### 3.1.1 The predicted dynamic interactions hypothesis

MultimerMapper uses graph theory to compare PPI patterns from simple dimeric predictions (2-mers) with those in more complex oligomeric predictions (N-mers). Consider a hypothetical system of three proteins: A, B, and C (Figure 1a). For the moment, assume that we can determine with certainty that an interaction between two of these proteins exists in a structural model, involving two specific surfaces (green checkmarks in Figure 1). The actual interaction prediction method is described in Section 2.4 Protein-Protein Interaction Detector, and the detection of interaction surfaces in Section 2.6 Contacts, Multivalency & Clustering: Interaction surface prediction.

**FIGURE 1:**
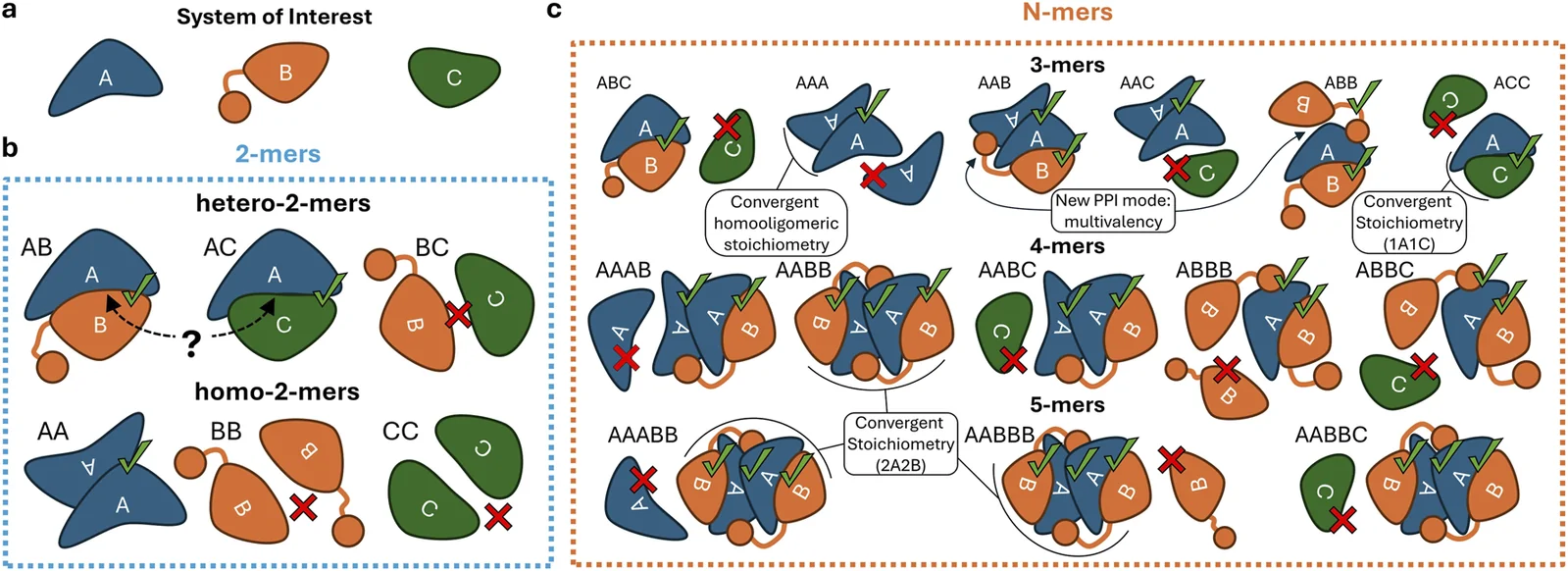
Hypothetical system with 3 proteins. **(a)** Domain architecture of proteins A, B and C. A and C are single domain proteins. B has two domains connected by a disordered region. **(b)** All possible dimeric predictions (2-mers) of the system. The presence or absence of interactions between proteins is highlighted with a green checkmark or a red cross, respectively. Protein A interacts with B and C through the same surface, indicated with a question mark. **(c)** Oligomeric predictions (N-mers) and the resulting interactions between subunits indicated with checkmarks and crosses. Key concepts described in this work and revealed by MultimerMapper’s structural analysis are highlighted inside white boxes. These include multivalency, convergent stoichiometry and convergent homooligomeric stoichiometry.

When all possible heterodimeric predictions are generated, we observe, in this hypothetical example, that A can interact with both B and C, while B and C do not interact with each other (hetero-2-mers in Figure 1b). Notice that the interaction surface of A in AB and AC is the same. Could both interactions occur simultaneously? We can manually analyze their surfaces, align both complexes and check for the steric hindrance. Instead, we predict the heterotrimer ABC and extract the PPI patterns in this new model (ABC 3-mer in Figure 1c). There, only the A-B interaction persists, while the A-C interaction “disappears” with respect to the 2-mers. Through this comparison, we will classify the A-B interaction as “static” (it is present in both 2-mer and 3-mer predictions) and the A-C interaction as “dynamic” (present in 2-mer but absent in 3-mer predictions). In this case, the dynamic behavior of the PPI stems from an underlying competition mechanism between B and C for the same surface.

To systematically detect and analyze these dynamic PPIs, we developed a computational framework based on graph theory (Pavlopoulos et al., 2011), with a set of rules to classify interactions and proteins based on their behavior (Figure 2a). By comparing the graphs generated from 2-mers and N-mers predictions, we can classify PPIs and proteins as static or dynamic, along with the dynamic type (positive or negative). In our example, as the A-C interaction was present in the 2-mers, but disappeared in the 3-mer, we classify its PPI as dynamic “negative”. This can happen when steric hindrances or conformational changes in higher-order complexes prevent certain interactions that were possible in simpler dimeric configurations (Redington et al., 2021). In contrast, a dynamic “positive” PPI will happen when the opposite is true: not present in 2-mers but present in N-mers. This can occur when the presence of additional proteins in a system creates new binding interfaces or induces conformational changes that promote interactions not observed in simpler dimeric predictions (Tompa, 2016).

**FIGURE 2:**
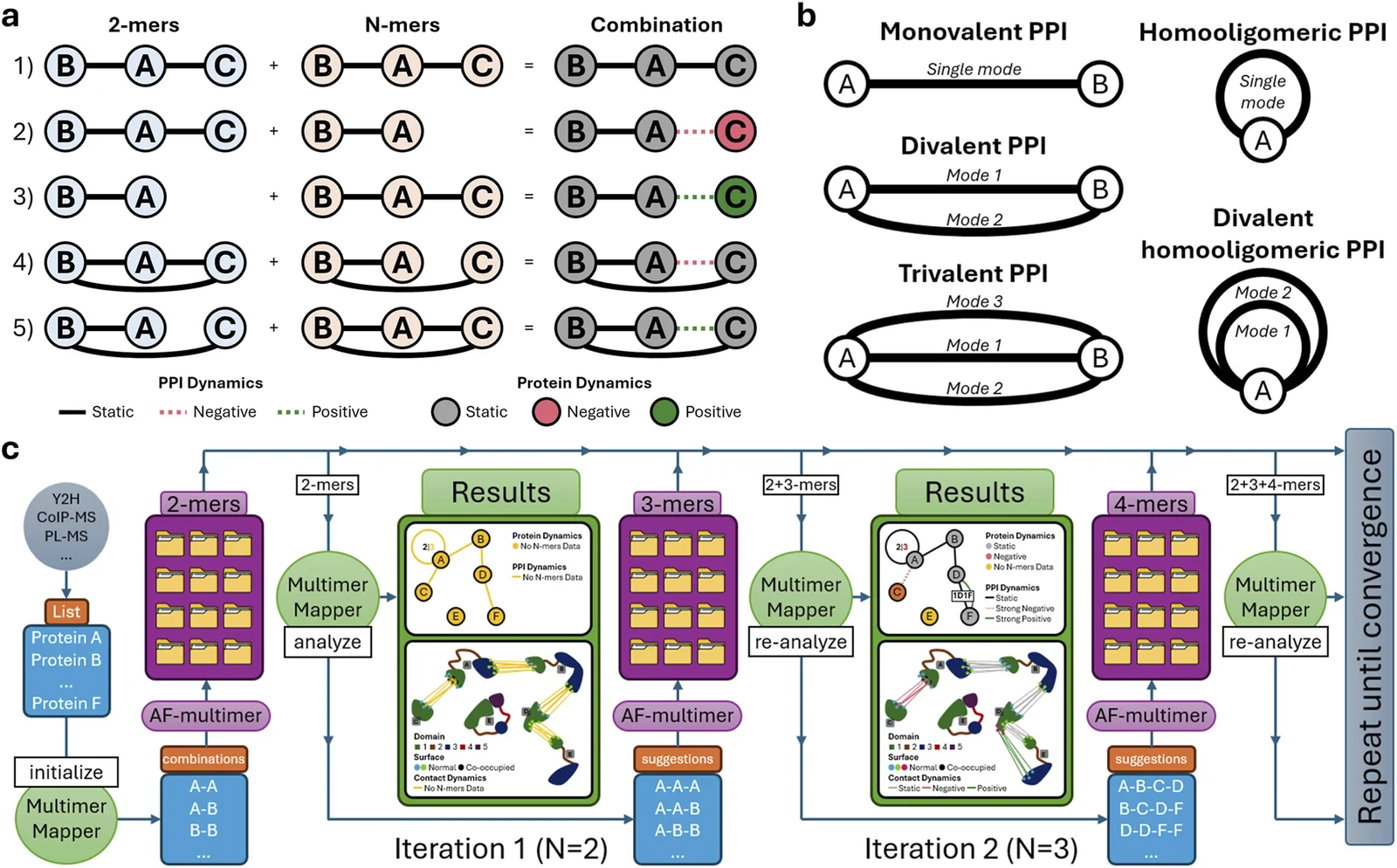
MultimerMapper’s PPI classification system and pipeline. **(a)** Logic of PPI and protein dynamics classification in a three proteins system. 1) Both interactions are present in both graphs. The proteins and interactions are static. 2) Both interactions are present in the 2-mer graph, but only the A-B edge is present in the N-mer graph. Therefore, C is a negative dynamic protein, and its interaction dynamics with B is negative. 3) The A-B edge is present in both graphs, and the B-C edge appears only in the N-mer graph. C is a positive dynamic protein, and its interaction dynamics with B is positive. This is a case of interaction activation induced by the presence of A. 4-5) Same case as 2 and 3, respectively, but involving a third interaction, A-C. In these cases, C is present in both combined graphs because it participates in a PPI that keeps this node in the final graphs. This makes protein C static, but not its interaction with B. **(b)** Graph representation of homooligomeric and multivalent interactions and their combinations. **(c)** MultimerMapper pipeline flow chart. A list of proteins with high probability to form complexes (*e.g.*, coming from yeast-two hybrid, Co-Immunoprecipitation or Proximity Labeling experiments) are the initial input for MultimerMapper (initialize). All possible 2-mer combinations are generated (combinations, blue box). The user predicts 2-mers using AlphaFold (2-mers, purple box). Resulting prediction are processed with MultimerMapper (analyze). This generates results (green box) and a new list of protein combinations (suggestions) that the user must predict (3-mers). Dimers and trimers are provided to MultimerMapper in a second iteration (N = 3), generating new results and new suggestions. The process is repeated until the convergence of the system.

With this approach, AF applies its physicochemical/co-evolutionary “knowledge” to decide which PPI prevails (if any) in the combinations, while we explore the outcome and map their behavior on a combined graph. However, when multiple N-mer models contain the same protein under many different conditions, the possibilities are no longer binary, as the same interaction can appear in one context and disappear in another. Thus, the actual rules consider these possibilities as a PPI dynamics spectrum, based on the frequency of the PPIs detected in the N-mers (see Section 2.8 PPI Graph Converter).

#### 3.1.2 Incorporation of homo-oligomerization and multivalent interactions

The framework also accounts for homo-oligomerization and multivalent interactions (multiple, independently binding interfaces within the same protein), two critical factors in determining complex stoichiometry (Nishi et al., 2013; Sipko et al., 2024). Homo-interactions are represented with self-connecting edges and multivalent interactions are represented as distinct paths between pairs of proteins (Figure 2b). These two can also occur in combination and can be classified by their dynamic behavior independently. The method to detect multiple interaction surfaces based on multivalency is described in Section 2.6 Contacts, Multivalency & Clustering: Interaction surface prediction.

Getting back to our thought example, after predicting the homodimers we only detect homooligomerization for A (homo-2-mers in Figure 1b). So, we can incorporate this information in a 2-mers PPI graph as an edge that self-connects A. Given that A is the only homooligomerizing protein, we explore the behavior of the homointeraction in different trimeric contexts by generating combinations containing two subunits of A and varying the third subunit (AAA, AAB and AAC in Figure 1c). For the homotrimer, we see that the self-interaction still occurs, but one of the subunits is left aside. In other words, this evidence suggests that the homotrimer is unlikely to be formed, while adding an extra subunit doesn’t disrupt the homointeraction. Interestingly, we notice that C does not interact with the homo-dimeric form of A in the AAC context. This suggests that something is happening with A when it homodimerizes, preventing the interaction with C (a conformational change in A?). We will see that MultimerMapper can detect conformational changes associated the presence or absence of certain proteins (see Section 2.5 Coordinate Analyzer and Section 2.8 PPI Graph Converter). On the other hand, B still interacts with the same surface on A in the AAB context, but a new interaction surface appears with another subunit of A, engaged via the smaller domain of B. This new interaction mode can be added to the graph as a different edge. This information provides insights into a potential regulatory role of protein C in the formation of an A-B complex.

#### 3.1.3 Iterative stoichiometric space exploration (SSE) and the convergence hypothesis

MultimerMapper works by suggesting combinations that users must predict to explore the stoichiometric space iteratively (Figure 2c). The system is initialized with a list of proteins, generating all possible 2-mer combinations. Once predicted and provided, the first iteration determines which pairs engage PPIs, suggesting 3-mer combinations. Predictions are given back to MultimerMapper to identify those 3-mers that form stable complexes. For now, let’s consider instability as having at least one red cross (Figure 1). The algorithm to detect stability and explore the stoichiometric space is described below in Section 2.7 Stoichiometric Space Exploration & Stoichiometry Prediction. Stable 3-mers are then chosen to generate new combinations (4-mers) that the user should predict for the next iteration, repeating the process. This strategy approaches gradually to the combination of proteins that we refer as “convergent stoichiometries” (Figure 1c), which generally match functional stoichiometries of the underlying complexes.

These suggestions maximize the identification of PPI dynamics, homo-oligomerization, multivalency and conformational changes within the system. It's important to note that MultimerMapper can’t automatically detect non-convergent geometries (*e.g.*, linear polymers like tubulin). In practice, users must identify them during the iterations, guided by MultimerMapper’s suggestions and visualizations, and remove them from the prediction to prevent infinite loops.

Computationally, the number of required predictions ( P ) is fixed for 2-mers and depends on the number of distinct polypeptides within the system ( n ):

$$P_{2-mers}=\frac{n\;\left(n+1\right)}{2}$$

Meanwhile, P will vary for N-mers, depending on the complexity of the system. In systems with no interacting pairs, N-mer predictions will be zero, while systems with potential for infinite growth (like linear polymers) could theoretically require an infinite P :

$$0\leq P_{N-mers}\leq \infty$$

Using the AF3 server (30 models per day), the complete pipeline took us less than 1 day for smaller systems, and up to 5 days for larger systems that converged at N = 10 (see Supplementary Material).

### 3.2 MultimerMapper architecture

The software is flexible, accommodates diverse datasets, detects errors introduced by the user, and identifies critical gaps in the data. It provides output both as computational objects and as user-friendly visualizations. The inner workings are illustrated in Figure 3.

**FIGURE 3:**
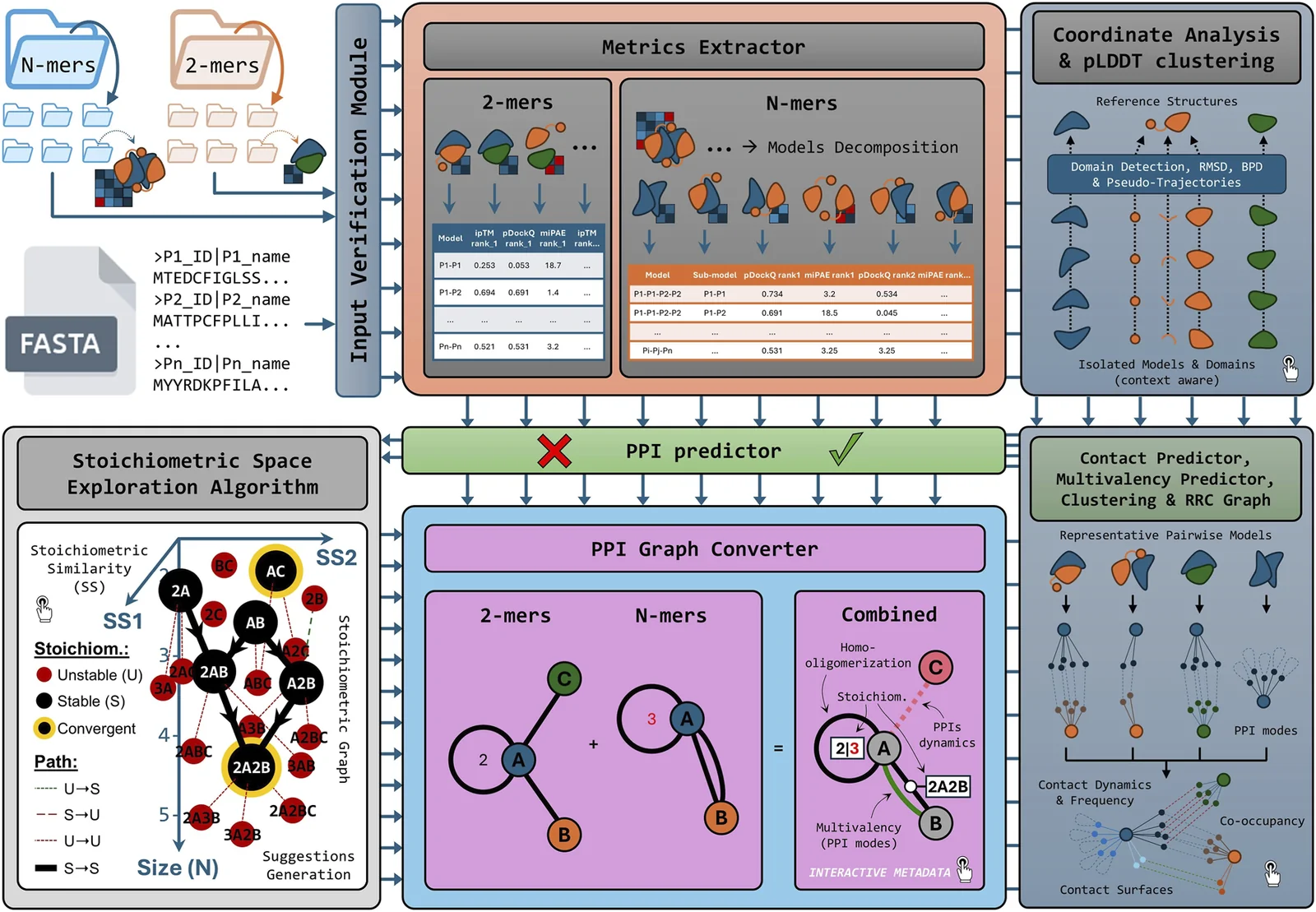
MultimerMapper main architecture and inner workings. Each core module of the software and their inner workings are represented as boxes. A description of how each module works can be found in the main text.

The minimum input requirements are:

1. FASTA file: sequences of distinct protein entities. Each header must include a unique identifier and name/symbol. For the initialization, this is the only input needed to generate the 2-mer combinations (Figure 2c).
2. A directory containing all 2-mer predictions.
3. Optionally, a directory with N-mer predictions. This enables the detection of dynamic interactions and convergent stoichiometries.

This setup supports the iterative pipeline, where users initialize the system with MultimerMapper, predict the suggested 2-mers dataset and then run MultimerMapper again to detect interacting protein pairs in a first iteration. If the objective is simply to detect PPIs between 2-mers, this step will be enough. However, users can predict the suggested combinations and re-run MultimerMapper with this additional information to identify dynamic interactions. It is worth noticing that modules from Figure 3 do not strictly represent the software’s architecture, but closely resemble the code organization. In the following sections, the functioning of each core module and the optimization of their parameters are described.

### 3.3 Metrics extractor

After a verification step to identify errors or inconsistencies (Input Verification Module, Figure 3), a first module pre-process 2-mers and N-mers separately (Metrics Extractor, Figure 3):

1. For 2-mers, it directly extracts all model metrics (pLDDT, pTM, ipTM, and PAE matrix), atomic coordinates and protein composition. Key metrics such as miPAE (minimum interaction PAE, see Section 2.4 Protein-Protein Interaction Detector) are computed, along additional metrics that form part of MultimerMapper’s output. Values are obtained for each of the five models predicted.
2. For N-mers, models are decomposed into its component pairs. For example, a 4-mer composed of two subunits of A and two of B (A_1_A_2_B_1_B_2_) is decomposed into all possible dimeric combinations (A_1_A_2_, A_1_B_1_, …, B_1_B_2_). Metrics and coordinates are then extracted/computed for each resulting sub-model and sub-PAE matrix for each of the five models, registering the protein composition of the pair and the original prediction.

The data is passed to downstream modules to continue the analysis process.

### 3.4 Protein-protein interaction detector

The next module (PPI Detector, Figure 3) takes the extracted metrics to determine which protein pairs of each prediction engage in PPIs. The optimization of the module is described below.

#### 3.4.1 Benchmark dataset generation

As no benchmark dataset for PPI prediction is available for trypanosomatids like for yeasts (Humphreys et al., 1979), a dataset was generated through manual curation of the literature. Pairs of trypanosomatid proteins with experimental evidence of direct interaction were retrieved, resulting in 157 pairs of interacting proteins (Supplementary Table S1). The negative dataset was generated using the following approach. Lists of proteins belonging to different eukaryotic complexes conserved in trypanosomatids were retrieved and labeled according to the complex name and subcellular compartment (Supplementary Table S2). Pairs of complexes with unrelated functions and separate subcellular compartments were defined, such that their components have a very low probability of having cross-interacting components. A pair of complexes was then randomly selected, and one protein from each complex was randomly chosen to generate a non-interacting pair. This procedure was repeated until 200 non-redundant pairs were obtained (Supplementary Table S3).

#### 3.4.2 Structure prediction and metrics extraction for benchmark

PPI benchmark dimers were predicted using both AF2m and AF3, randomly selecting 157 negatives with less than 2000 amino acids to equilibrate both datasets. Five models were predicted per dimer, ranking the models based on ipTM values. Associated metrics were extracted (pLDDT, pTM, ipTM, PAE) and two additional interaction metrics were computed: pDockQ, widely used and validated for interaction prediction (Bryant et al., 2022a); and miPAE, which simplifies the PAE matrix by focusing on interaction regions. Now calculated by default by the AF3 server (Abramson et al., 2024), miPAE identifies the minimum value in the PAE matrix for residue pairs across different chain pairs. The resulting data can be found in Supplementary Material S1.

#### 3.4.3 Cutoffs optimization

To identify which metric and what cutoff maximizes PPI prediction sensitivity and minimize non-specificity, we performed a receiver operating characteristic (ROC) analysis using the interaction metrics (pDockQ, ipTM and miPAE). For each metric, we tested the effect of the number of models (N_models_) required to exceed the variable metric cutoff to consider an interaction. The resulting ROC plots can be seen in Supplementary Figure 2. We then extracted the cutoffs at discrete false positive rates (FPR) and measured its sensitivity (Figure 3 and Supplementary Material S2).

We observed that the best metric to predict pairwise PPIs is miPAE for both methods (Figure 4a), as it derives in the highest sensitivities, followed by the ipTM (Figure 4b) and then the pDockQ (Figure 4c). The same conclusion can be obtained by analyzing the area under the ROC curves (Supplementary Figure 3), being higher for miPAE (miPAE: AF2 = 0.847, AF3 = 0.834/ipTM: AF2 = 0.813, AF3 = 0.815/pDockQ: AF2 = 0.804, AF3 = 0.791). In general, results for AF2 and AF3 were similar, and at FPR = 0.05, AF2m and AF3 have similar sensitivity profiles, but they varied in the obtained miPAE cutoffs (Supplementary Material S2).

**FIGURE 4:**
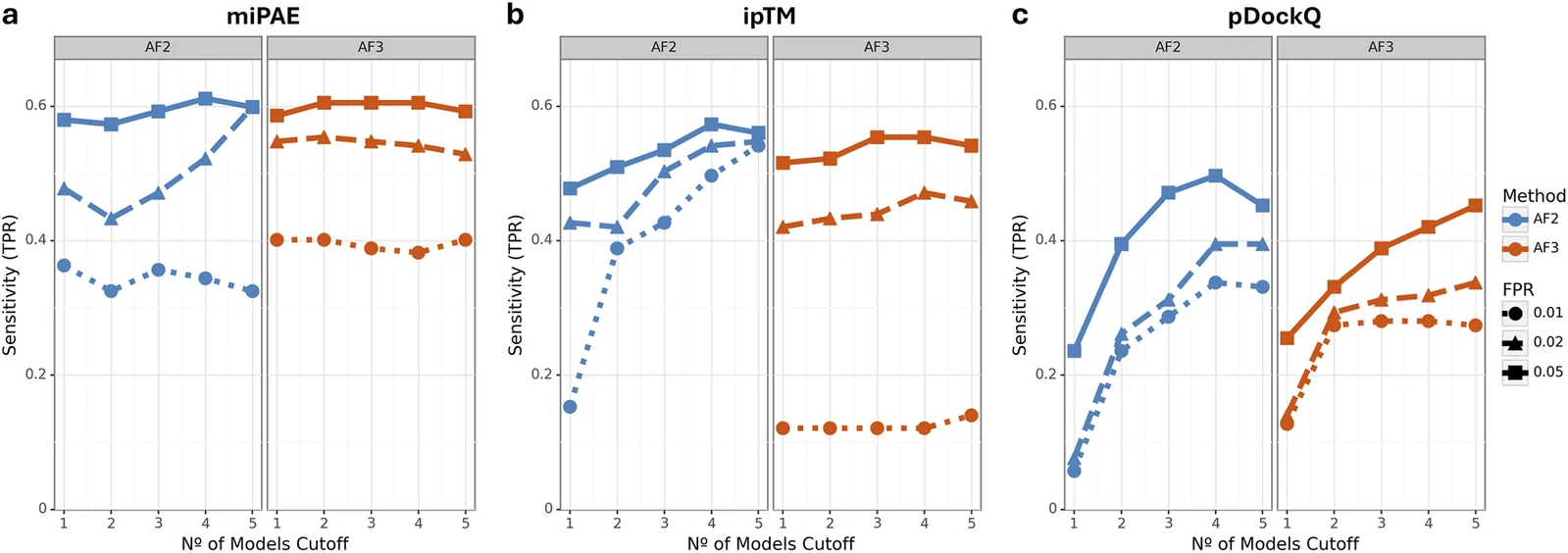
PPI prediction sensitivity profiles for different cutoff combinations and methods. Measured sensitivity using AlphaFold2-multimer (AF2) and AlphaFold3 (AF3) at different FPR levels (0.01, 0.02 and 0.05), N_models_ cutoffs and metrics. Profiles using the interaction metrics miPAE **(a)** ipTM **(b)** and pDockQ **(c)**.

By default, we’ve set the cutoffs of miPAE≤9.7 Å for AF2 and miPAE≤7.5 Å for AF3, with $N_{\text{models}}=4$ , which resulted in approximately 61% sensitivity at FPR = 0.05 for both methods (Figure 4a). During MultimerMapper executions, this criterion is applied directly for 2-mers. For N-mers, instead, it is applied to detect PPIs between any decomposed pair containing the target protein entities. For example, if a complex with 6 chains contains 2 subunits of the target protein A and 1 subunit of target B, for any given model, we consider that it has surpassed the cutoff if any miPAE of the two possible decomposed pairs for A-B surpass the cutoff. Then, if 4 models surpassed this cutoff, we consider that A and B interact in the corresponding prediction. Then, at a later stage, within the execution, MultimerMapper identifies which of these chains are in physical contact (see Section 2.6 Contacts, Multivalency & Clustering: Interaction surface prediction). In other words, the PPI Detector only identifies PPIs between protein entities within an N-mer combination, not between which chains.

### 3.5 Coordinate analyzer

This module (Coordinate Analyzer, Figure 4) processes the atomic coordinates, intramolecular PAE submatrices and pLDDTs of isolated chains from all models to evaluate how the protein conformation behaves depending on the modelling context. It first extracts a reference structure for each protein by selecting the chain with the best average pLDDT, which is used as reference point for several computations (Shor and Schneidman-Duhovny, 2024). This module then:

1. Identifies well-structured domains and disordered segments by clustering the intramolecular PAE matrix of the reference structure (Croll, 2024). It also identifies functional domains using InterProScan (Paysan-Lafosse et al., 2023).
2. Calculates the root mean square deviation (RMSD) of isolated chains/segments against a reference.
3. Computes pseudo-trajectories by sorting the chains/segments by increasing RMSD.
4. Computes metrics and visualizations to understand how and why trajectories evolve.
5. Clusters the chains by their pLDDT profiles and analyzes the enrichment levels of different protein entities on each cluster. For an explanation about pLDDT clustering, see the Supplementary Material.

All computations are aware of the modelling context that produced each isolated chain/segment, which is used to associate variations in metrics with the presence or absence of specific proteins. The obtained data is then processed by several interconnected modules downstream.

#### 3.5.1 Domain detection

A domain detection algorithm identifies well-structured and disordered segments. It analyzes the intramolecular PAE matrix to partition proteins into regions of high internal structural coherence, offering flexibility through automatic, semi-automatic, and manual modes, allowing users to easily get fine-tune results. The software generates interactive representations in real-time, aiding in the interpretation and validation of domain assignments (Supplementary Figure 4).

In semi-automatic mode, users can interactively adjust a resolution parameter (default 0.075) to optimize domain definitions for each protein. These optimized resolutions are stored and can be used across MultimerMapper’s iterations. The algorithm incorporates a loop removal step, ensuring that small, potentially flexible regions are appropriately incorporated into larger domains, while larger loops are considered as unstructured domain boundaries. Lower resolution values will split proteins into fewer domains, and *vice versa* (Supplementary Figure 4).

It’s worth noting that these segments we referred as domains aren’t necessarily equivalent to the functional domain definition (Kelley and Sternberg, 2015). Rather, they represent continuous segments of proteins that exhibit similar behavior in the intramolecular PAE matrix of the reference structure. This approach allows for the separation of proteins into compact, well-structured defined segments and more flexible or disordered regions. This provides a rational framework to explore how metrics and conformations vary for these segments instead of just whole proteins, increasing the detail of the posterior analysis. For functional domains identification, the InterProScan REST API is automatically queried using the protein sequences (Paysan-Lafosse et al., 2023).

#### 3.5.2 RMSD calculations and pseudo-trajectories

AlphaFold has demonstrated the ability to predict conformational variation for monomers when the input MSA is sampled (M et al., 2024). We reasoned that our methodology also generates different MSAs for each combination, but sampling at the multimeric level. To explore conformational heterogeneity as product of the contextual sampling, MultimerMapper constructs pseudo-trajectories by sorting the isolated conformations in ascending RMSD order relative to a reference. This is performed for both entire proteins and individual domains, providing a rich dataset for understanding structural variability and flexibility. An example of the resulting “RMSD pseudo-trajectories” can be seen in Supplementary Video S1. When the conformational states along the pseudo-trajectory is analyzed in parallel to the context, RMSD, radius of gyration (ROG) and pLDDT profiles, they offer deeper insights into how proteins respond to different modeling contexts (see below).

#### 3.5.3 Bias in partner distribution (BPD) analysis

To understand how and why these trajectories evolve, MultimerMapper computes per-frame features that can be interpreted with the conformational states. These are provided as interactive plots (Figures 5a–f) in parallel to an integrated pseudo-trajectory visualizer to reproduce the pseudo-trajectory (Supplementary Figure 5). This setup facilitates the exploration of the structures at each frame along the features without relying on external software (Supplementary Material S3).

**FIGURE 5:**
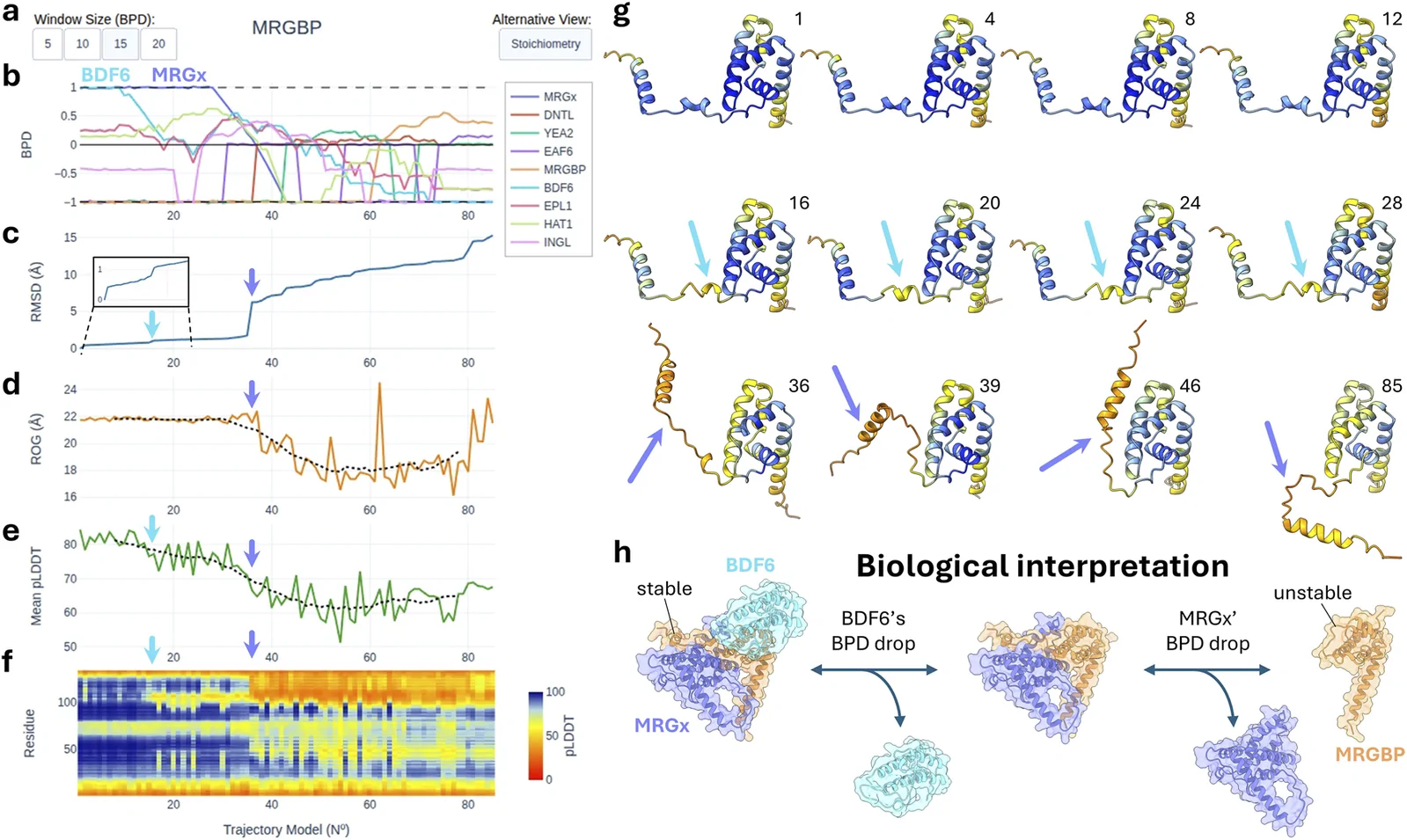
Data and visualizations generated by the coordinate analyzer module. Example data generated for the protein MRGBP from *T. brucei* (TriTrypDB ID: Tb927.8.5320) in the context of the NuA4 protein complex system (Staneva et al., 2021). **(a)** Interactive buttons allows switching between stoichiometry visualizations (right) and window size using BPD (left). **(b)** BPD versus frame (Trajectory Model N°) plot. Each line represents the BPD measured for each partner along the pseudo-trajectory. BPD values for BDF6 (light blue) is maximum at the beginning of the pseudo-trajectory, then drops to approximately zero between frames ∼20-50, and then decreases to −1 at the end. For MRGx (violet), BPD is also maximum at the beginning, but drops drastically after BDF6’s BPD drops to 0, reaching −1 around frame 40. **(c)** RMSD versus frame plot. Sudden increases in RMSD (arrows) match the inflection points seen in the BPD plot for BDF6 and MRGx. The inset shows the zoom between frames 1 and 25. **(d)** ROG versus frame plot. ROG values are constant until BPD values for MRGx become negative, diminishing ∼4 Å. The dashed line indicates the average ROG across each window. **(e)** Mean pLDDT versus frame plot. The average quality of the model drops slightly when BDF6’s BPD drops, but drops much more when MRGx’ BPD drops. The dashed line indicate the average mean pLDDT across each window. **(f)** pLDDT heatmap for MRGBP across the pseudo-trajectory. The y-axis indicate the residue position. Changes in per-residue pLDDT patterns match the variations in RMSD, ROG and BPD (arrows). Pseudo-trajectory frames after the purple arrow show low pLDDT values, particularly for the C-terminal residues. **(g)** Sample MRGBP conformations across the pseudo-trajectory, colored by pLDDT. The numbers indicate the pseudo-trajectory model number (or frame). Arrows indicate the structural changes observed compared to the reference (frame 1). The light blue arrow points to a small change in an alpha helix. The purple arrow indicates the oscillation of the C-terminal segment. **(h)** Biological interpretation of the data using the stable stoichiometries identified by MultimerMapper. When BDF6 “detaches” from the heterotrimer, just a small variation is observed in MRGBP’s structure. When MRGx “detaches” from MRGBP, the C-terminal segment of MRGBP becomes unstable. The “release” of MRGx only happens if BDF6 “detaches” from MRGBP first. This can also be interpreted in the opposite sense (assembly instead of disassembly).

The RMSD, besides being used to generate the pseudo-trajectory, provides a good measure of the conformational variation along the pseudo-trajectory. Sudden changes in RMSD may indicate major conformational changes occurring at specific points (Figure 5c). Another informative metric is the Radius of Gyration (ROG), which assess the overall compactness of the structure throughout the pseudo-trajectory (Ahmed et al., 2020). Variations in ROG may indicate transitions between more compact and extended conformations, reflecting potential structural rearrangements or binding events (Figure 5d). Mean pLDDT provides insights into average changes in overall local confidence (Jumper et al., 2021). Abrupt changes in mean pLDDT may indicate the engagement (or disengagement) of an interaction at specific points that affect confidence, whereas stable high values can suggest structural stability along the whole pseudo-trajectory (Figure 5e). Additionally, a per-residue pLDDT heatmap for the pseudo-trajectory is provided to pinpoint specific regions responsible for confidence variations (Figure 5f).

To correlate these variations with the presence or absence of specific partners, we developed a novel measure that we call Bias in Partners Distribution (BPD). This metric evaluates whether certain proteins are preferentially enriched or depleted at specific points of the pseudo-trajectory. BPD describes the local frequency of each protein in a sliding window relative to its overall frequency. A bias score of 1 indicates that a protein is strongly enriched in a given window, while a score of −1 means it is depleted. A value around 0 suggests a random distribution, meaning the presence of the protein is not significantly biased within the window (Figure 5b). This helps to identify whether specific partners are associated with particular conformational states, providing insights into potential binding preferences, induced fit mechanisms or assembly paths.

For example, the protein from Figures 5a–f is MRGBP from *Trypanosoma brucei*, analyzed within the context of the NuA4 complex protein system (Rodríguez Araya et al., 2023; Staneva et al., 2021). Previous molecular dynamics simulations and experimental data demonstrated that MRGBP binds sequentially to the proteins MRGx and BDF6 to form the conserved eukaryotic subcomplex TINTIN. MRGBP is too unstable to interact with BDF6 in its free form; instead, it first binds to MRGx, which stabilizes its C-terminal segment and enables its subsequent interaction with BDF6 (Rodríguez Araya et al., 2023). This same sequential binding pattern emerges in our pseudo-trajectory analysis, where BPD scores indicate a high bias for both MRGx and BDF6 in early frames, followed by the sequential decrease of BDF6 BPD values, and then MRGx (Figure 5b). The initial depletion of BDF6 (frame at which the BDF6 gets a BPD of zero) is associated with a small increase in RMSD and a small decrease of pLDDT (light blue arrows in Figures 5c–g). At the conformational level, the change occurs in the first section of the extended segment of MRGBP (light blue arrows in Figure 5g). After the depletion of MRGx, an abrupt change in conformation, reduction of the ROG and drop in confidence scores can be observed (purple-blue arrows). This mirrors previous molecular dynamics and experimental observations (Rodríguez Araya et al., 2023), but now captured through a modeling-based approach (Supplementary Video S2). A scheme of the biological interpretation can be seen in Figure 5h.

Another useful visualization to identify trends in protein context associated with specific conformations is the variation in stoichiometric coefficients for each protein along the pseudo-trajectory. This is complementary to the BPD and gives information about the specific amount of each protein in the frames of the pseudo-trajectory. The interactive version has buttons to switch between the Stoichiometry and BPD visualizations (Figure 5a).

This method was tested using seven non-trypanosomatid systems known for containing context-dependent conformational changes. In six of them the expected conformational changes and their association with the expected proteins or contexts were correctly predicted, by using the BPD as the primary readout and the Stoichiometry View as complementary evidence for cases with more complex stoichiometries (see Supplementary Material “Context-Dependent Conformational Change Prediction Testing” for details).

### 3.6 Contacts, multivalency & clustering: interaction surface prediction

Several modules (Contact predictor, Multivalency Predictor, Clustering and RRC graph, Figure 3) analyze the interaction surfaces between protein pairs. Together, they predict the intermolecular residue-residue contacts (RRC), predicts multivalency, clusters the predicted RRC matrixes to identify distinct PPI modes and generates RRC graph representations. Individual intermolecular contacts are also classified as dynamic or static, simultaneously tracking residues involved in multiple contacts that could potentially cause steric hindrances (surface co-occupation). Interactive representations to explore the results are also generated.

#### 3.6.1 Intermolecular residue-residue contacts prediction

Interchain RRCs are predicted for 2-mers and decomposed N-mer pairs that were detected engaging PPIs in their corresponding predictions (see Section 2.4 Protein-Protein Interaction Detector). For those that PPI detection resulted negative, RRCs are considered null. The method combines information from the interchain PAE and interchain residue distance matrixes to predict which residue pairs are in contact (Supplementary Figure 6). Briefly, a residue pair is considered in contact if they are sufficiently close (less than 8 Å) and the predicted error is lower than the PAE cutoff used for PPI detection.

We have compared the results of this method with COCOMAPS 2.0 (Chawla et al., 2025) on four PPI modes from the stoichiometry inference benchmark. Leaving aside the fact that COCOMAPS 2.0 classifies the physicochemical nature of each contact, the predicted RRC patterns were very similar between both approaches (Supplementary Figures 7–11).

#### 3.6.2 Multivalency prediction

Once identified which chains are in contact inside each model, MultimerMapper predicts which pairs of protein entities engage multivalent interactions. This is done by counting how many chains are simultaneously in contact with more than one subunit of the corresponding opposite (Supplementary Figure 12a). To distinguish real from spurious multivalency, different metrics derived from the counts were considered and tested against a benchmark dataset curated from the literature, consisting of multivalent and monovalent protein pairs (Supplementary Table S4 and Supplementary Material S4). The best performant metric was the fraction of multivalent chains (Supplementary Figures 12b,c), obtaining a sensitivity of 73% and a specificity of 93% using a cutoff of 0.2.

#### 3.6.3 Contact matrix clustering

For multivalent protein pairs, MultimerMapper predicts the number of distinct PPI binding modes (see Figure 2b) by clustering of their pairwise contact matrixes. Briefly, an RMSD distance matrix is computed between all decomposed pairwise models, considering only the alpha-carbon coordinates of residues involved in contacts, and used as input for hierarchical clustering, where each resulting cluster represents a distinct interaction mode (Supplementary Figure 13).

When individual RRC matrixes are merged into clusters, we can also obtain an average representation of the binding interface of each mode. It allows computing how frequent each interaction mode is represented in the N-mers, and how frequent are the individual contacts. The implementation is also aware of which combinations of proteins contributed more to each cluster and, by comparing which context produced which contact mode, we can statistically associate the presence or absence of specific partners with particular PPI modes (see Section 2.8 PPI Graph Converter). Moreover, individual contacts can also be classified into static, dynamic positive and dynamic negative.

This module also identifies a representative structure for each binding mode by selecting the pairwise model with the best contact matrix overlap to the average contact matrix of the cluster. For example, we processed the system composed of the protein entities SEC13 and SEC31 until convergence. Figures 6a,b show the representative structures obtained for the pairs SEC31-SEC31 and SEC13-SEC31, respectively. These two proteins form the outer coat of the core eukaryotic complex COPII, a cage-like complex involved in the budding of vesicles that is essential for secretory pathways (Zanetti et al., 2012). The identified clusters reflect the flexibility of SEC31 and heterogeneity of interactions, something that has been experimentally observed, which is key for the function of the cage (Whittle and Schwartz, 2010). SEC31-SEC31 Cluster 0 and 2 representatives are comparable to previously observed conformations of SEC31, while cluster 1 and 3 representatives seem to be transition conformations between cluster 0 and 2 (Figure 6a). Meanwhile, the clusters for SEC13-SEC31 show how SEC31 can bind SEC13 using only its C-terminal, only its N-terminal or with both simultaneously (Figure 6b). Furthermore, contact cluster information can be analyzed in parallel with the pseudo-trajectory of SEC31, which captures this conformational transition in more detail (Figure 6c and Supplementary Video S3).

**FIGURE 6:**
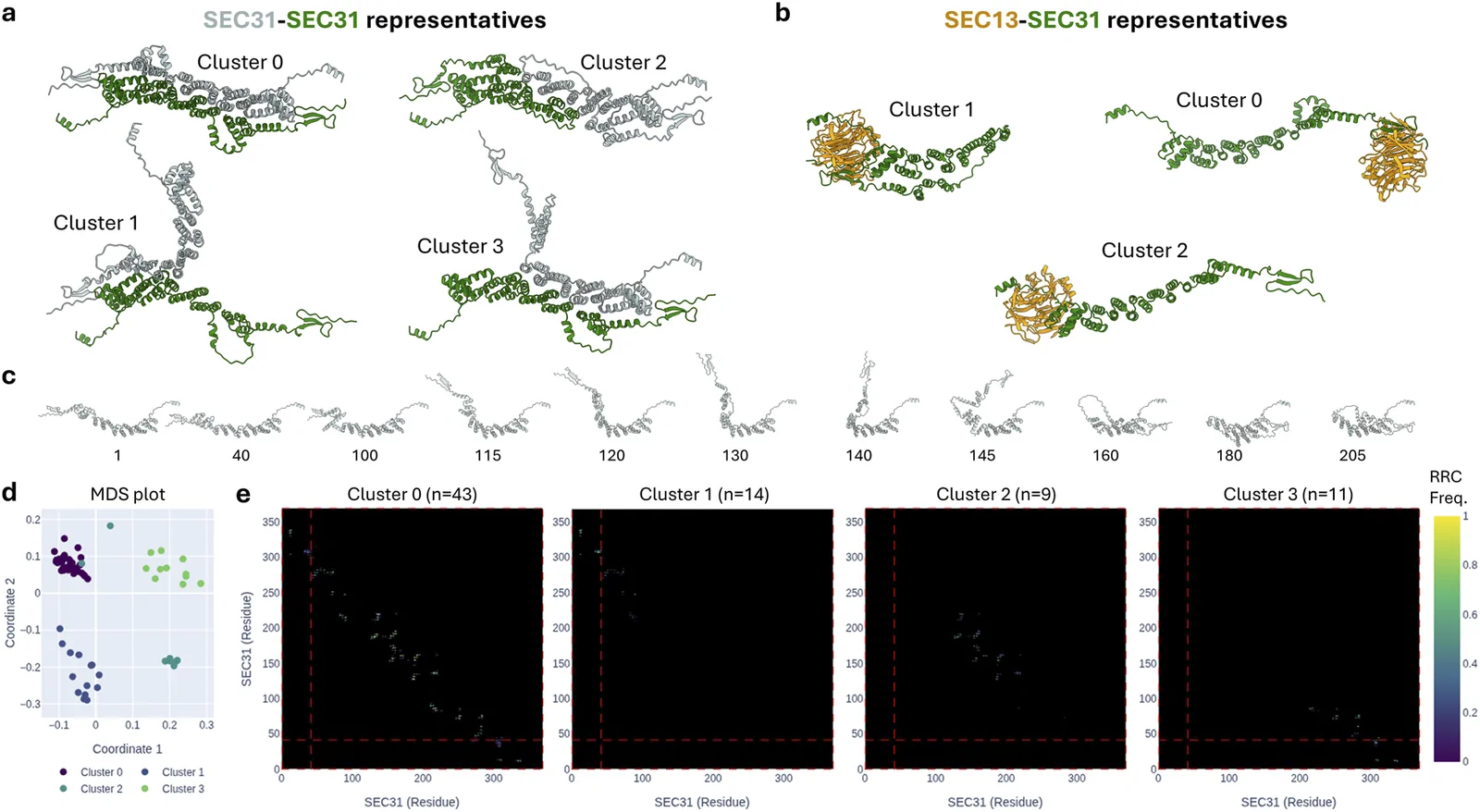
Contact matrix clustering. Results of the contact matrix clustering algorithm for the system composed of the protein entities SEC13 (ID: Lsey_0020_0440) and SEC31 (ID: Lsey_0451_0040) from *Leptomonas seymouri*. **(a)** Representative structures assigned for the pair SEC31/SEC31 clusters. **(b)** Representative structures assigned for the pair SEC13/SEC31 clusters. **(c)** Pseudo-trajectory models showing the conformational variation of SEC31. Numbers indicate their corresponding frames in the pseudo-trajectory. **(d)** MDS plot colored by contact cluster for the pair SEC31/SEC31. Each marker represents a decomposed pairwise model. Coordinates 1 and 2 are low-dimensional embeddings that place models so that those closer have small RMSD and those far apart have large RMSD. **(e)** Corresponding average contact matrixes for each cluster from panel **(a)**. The number of pairwise models assigned to each cluster is shown between brackets (n) and the frequency of each RRC in the cluster is color coded. Segmentation boundaries determined by the domain detection algorithm are indicated with dashed lines.

Also, a multidimensional scaling (MDS) analysis integrates the RMSD distances, describing the relationships between pairwise models (Figure 6d), along the corresponding average contact matrixes of each cluster (Figure 6e). All these outputs are provided as interactive visualizations that users can explore to understand the contextual behavior of proteins (Supplementary Figure 14).

On the other hand, monovalent pairs are considered as interacting through a single binding mode, and their contact matrixes are grouped into a single cluster.

#### 3.6.4 Residue-residue contacts graph representation

To visualize what are the available contact surfaces between proteins, a 3D graph representation of the RRC clusters is generated and included as default output (Supplementary Figure 15). It represents each protein in 3D space using the reference structure, with individual RRCs depicted as lines that connect the residue centroids that were predicted to be in contact. The thickness of these lines is proportional to the frequency of the RRCs and are colored following the rules described in Figure 2a, but at the residue-level (static in gray, dynamic negative in red and dynamic positive in green). A force field algorithm is executed to orient the surfaces properly and avoid collisions. The visualization is interactive, with different color schemes (by pLDDT, by domain, by residue, etc.), protein styles (cartoon, spheres, etc.) and other customizable features available to explore the predicted RRCs between proteins.

### 3.7 SSE and stoichiometry prediction

A core module (Stoichiometric Space Exploration Algorithm, Figure 3) suggests combinations, finds stable and convergent stoichiometries and decides when overall convergence has been reached. Besides predicting stoichiometries, this module is key to generate a rich dataset of models coming from different contexts that can be decomposed and analyzed by the rest of the software. Additional functionalities provide aid in the identification of possible stoichiometries.

By combining these methods, we were able to distinguish unequivocally the expected stoichiometry in 60% of the cases on a benchmark dataset of complexes from multiple species, subunit composition and symmetries, randomly sampled from the Complex Portal database (Balu et al., 2025). In 80% of the cases, we were able to identify the expected stoichiometry at least forming a stable complex (see Supplementary Material “Complex Stoichiometry Inference Testing” section).

#### 3.7.1 The SSE algorithm

A schematic representation of the how the algorithm works for a hypothetical system of two proteins, A and B, that form a complex with a 2A2B stoichiometry is shown in Figure 7a. After providing the 2-mers (first iteration), MultimerMapper decides which pairs form stable complexes. These pairs are considered as seed stoichiometries to explore the stoichiometric space through the construction of a directed graph, where stable stoichiometries (parents) can generate new stoichiometries (children) that must be predicted for the next iteration. Briefly, 2-mer stoichiometries are considered stable if they are predicted to be interacting (see Section 2.4 Protein-Protein Interaction Detector), while N-mer stoichiometries are considered stable when multiple independent structural models consistently show all subunits interconnected trough RRCs into a single contiguous assembly (Figure 7b). Children stoichiometries are built by taking the combination of proteins in the stable parental stoichiometry and adding one subunit of each protein entity to each new child. Meanwhile, unstable stoichiometries do not generate children. With this setup, the directed graph grows by 1 unit at each iteration, exploring only informative combinations that have potential to be stable. Each stoichiometry can have multiple parents, and the biggest stable stoichiometries found at each branch is considered a convergent stoichiometry. The rationale behind this algorithm is that the proteins in convergent stoichiometries have all of their potential interaction surfaces engaged, and adding new subunits would result in free subunits.

**FIGURE 7:**
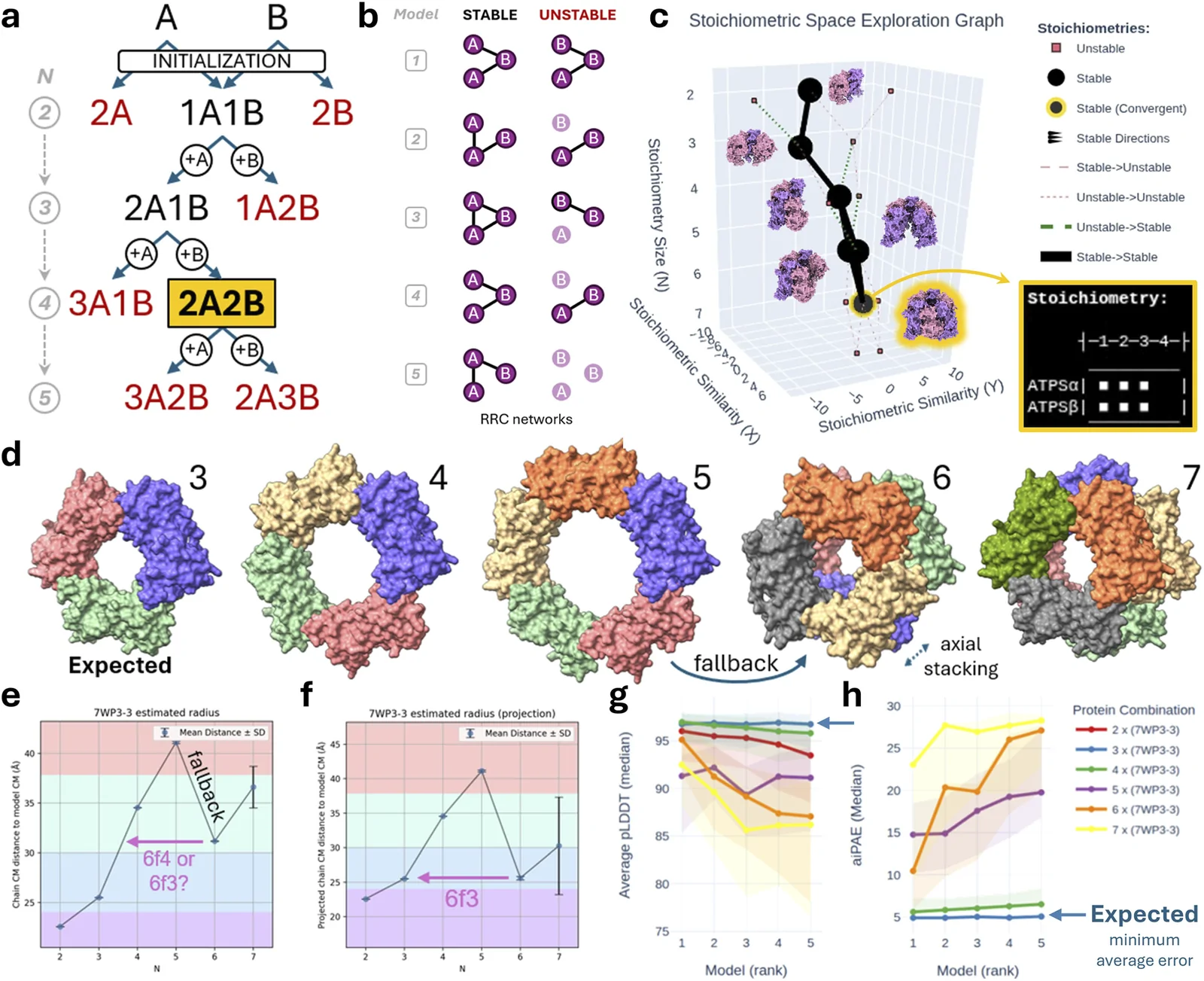
Stoichiometry prediction methods. **(a)** Schematic representation of the stoichiometric exploration algorithm for a simple system of 2 protein entities that converges in a 2A2B stoichiometry (gold inset). In the initialization, all 2-mers are computed. At the first iteration (N = 2) MultimerMapper decides which pairs are stable. Unstable stoichiometries (red) do not generate children. Stable stoichiometries (black) generate children stoichiometries by adding +1 of each protein entity. These children stoichiometries (suggestions) must be predicted and included in the next iteration (N = 3). The process is repeated until all the terminal stoichiometries from the hierarchies are unstable (convergence). **(b)** Definition of stability for the 2A1B 3-mer. For each model, a RRC network is built. Stable N-mers generate fully connected networks reproducibly (left). Unstable N-mers systematically leave chains outside the RRC network (right). **(c)** Interactive SSE graph for the mitochondrial ATP synthase system of *T. brucei*, subunits alpha (ID: Tb927.7.7420) and beta (ID: Tb927.3.1380). Each marker represents a stoichiometry (or protein combination). Stable stoichiometries are represented as black circles and unstable as small red squares. The convergent stoichiometry is represented with a golden outline and the metadata with the identified stoichiometry is shown in the inset. Lines represent the hierarchy between stoichiometries from different N values. The structures of the stable stoichiometries are shown next to their corresponding markers. **(d)** Rank 1 structures for PCNA from *Leishmania donovani* (ID: LdBPK_151500.1). The expected stoichiometry structure is highlighted (N = 3), along with the N value at which the symmetry fallback is detected. **(e)** Estimated radius of models shown in panel **(d)**. Bars represent the standard deviation (SD). **(f)** Estimated radius of models shown in panel d using 2D projection. **(g)** Average pLDDT confidence profile for different homooligomer sizes of PCNA. An arrow points to the expected stoichiometry confidence profile. **(h)** Average interchain PAE (aiPAE) profiles. The arrow indicates the profile for the expected stoichiometry, which has the lowest average interchain error.

After every MultimerMapper run, an interactive 3D visualization showing the evolution of the SSE is provided, allowing users to visually explore the results of the algorithm. For example, Figure 7c shows the results for the system composed of the mitochondrial ATP synthase, alpha and beta subunits, from *Trypanosoma brucei* (Montgomery et al., 2018).

By default, all possible +1 additions of each protein entity are explored for each stable stoichiometry, but we included specific parameters to control how stringent the stoichiometric space is explored (Table 1). For example, only protein entities within the same PPI network of the components that form the parental stoichiometry can be added to generate children stoichiometries. To illustrate, suppose that the stoichiometry 3B has been detected as stable in a system of 5 protein entities (A, B, C, D and E). A, B and C have been detected interacting with each other, while D and E interact in a different subnetwork. In this case, 3B will generate only the children 1A3B (3B+1A), 4B (3B +1B) and 3B1C (3B+1C). Additionally, for systems were the involved complexes grow by the addition of dimers and not just isolated monomers, a dedicated parameter can control the order of increments. The clearest example is the nucleosome, that assembles physiologically through the addition of histone dimers (Chen et al., 2014). AlphaFold seems to replicate this behavior, as the nucleosome system converges in the isolated dimeric complexes if this parameter is set to add the default increments of 1 (Supplementary Figure 16a). In contrast, MultimerMapper finds the expected convergent stoichiometry of the nucleosome (2:2:2:2) when the parameter is set to work using increments of 2 (Supplementary Figure 16b). This parameter works by generating children stoichiometries that not only adds +1 of each protein but also adds +2, using the stoichiometries of the stable 2-mers to each stable stoichiometry. It can be set to any integer, for example, to add up to +3 increments (adds stable 2-mers and 3-mers) or +4 (2, 3 and 4-mers), to cover other potential types of complex assemblies (see methods for more information), but computational cost must be considered when modifying this parameter.

#### 3.7.2 Homooligomer fallback detection

For some cyclic homooligomers, MultimerMapper can predict stable stoichiometries with structures that surpass the expected size. In these cases, we noticed that AlphaFold predicts high confidence structures of increasingly bigger cyclic structures beyond the expected assembly. However, there is a point at which they experience a “fallback” to a more compact architecture of stack repetitions of the expected stoichiometry. For example, the conserved protein PCNA from *Leishmania donovani* is known to form rings of size 3 or hexamers formed by two stacked trimers (Yadav et al., 2017). However, MultimerMapper also predicts to be forming stable 4 and 5 subunits rings. At N = 6, it “falls back” to the expected structure composed of the two stacked elements of the structure predicted at N = 3 (Figure 7d). We found similar patterns in other cyclic homooligomeric assemblies (Supplementary Figure 17).

To identify this behavior, we build an algorithm that detects significant drops in the estimated radius of the consecutive assemblies. If a fallback is identified, the algorithm searches for a matching radius using the confidence interval of the falling back model’s estimated radius. As seen in Figure 7e, the interval does not cover any of the previous radius. However, by applying a 2D projection before estimating the radius at each N value, the estimated projected radius confidence interval covers perfectly the estimated projected radius at N = 3 (Figure 7f and Supplementary Figure 1). This information is provided using the nomenclature YfZ (Y falls back to Z), where Y is the N value at which the fallback occurs and Z is the one to which Y falls back.

#### 3.7.3 Pairwise confidence profiles

Another useful observation is the tendency of average confidence metrics to peak at the expected stoichiometry. For example, Figure 7g shows the per-rank average pLDDT for PCNA in different stoichiometric combinations, where the average pLDDT profile is maximum for the expected stoichiometric combination (N = 3). Something similar happens for the average interaction PAE (aiPAE). In this case, the average error is minimum at N = 3 (Figure 7h). These confidence profiles help to easily determine which protein combinations generate the pairwise interactions with the best confidence metrics, but also can help to decide which is the most probable stoichiometry for homooligomeric or multivalent pairs.

### 3.8 PPI graph converter

This module (PPI Graph Converter, Figure 3) integrates the interaction information retrieved by other modules into graph representations, following the rules described in Section 2 (Figures 2A,B). Additional statistical analyses are performed to explore the significance of associations between the presence or absence of certain partners with specific PPI patterns and conformational states.

The method for predicting PPI dynamics was tested using 12 non-trypanosomatid systems with known PPI dynamics, correctly predicting the expected PPI dynamics in 8 of them (see Supplementary Material “Context-Dependent Dynamic Interaction Prediction Testing” section for details).

#### 3.8.1 PPI network representation

MultimerMapper creates a network where vertexes represent protein entities and edges represent PPI modes. Proteins that don’t interact with any protein are left outside the network. For each PPI mode, it analyzes in which context it was detected, its relative frequency and highlights whether the PPI wasn’t tested. The PPI classification system is summarized in Table 2. For visualizations, a different style is assigned to each PPI dynamic type, representing PPI frequency as edge width. This allows to easily identify important interactions, interactions that tend to appear (dynamic positive) or disappear (dynamic negative) in more complex scenarios and in which frequency. By default, indirect interactions are removed. An interactive visualization is provided to explore useful metadata about the proteins and their interactions, like the exact frequency or the number of pairwise models that were detected interacting through particular PPI modes. For interactions with the potential to form higher order subassemblies (like homooligomers and multivalent interactions), the stability of the different combinations is shown using insets that can be turned on and off.

**TABLE 2:** Extended PPI classification system.

Style	PPI dynamics	PPI in 2-mers	PPI in N-mers	N-mers PPI frequency (f)	N° of contacts In N-mer
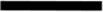	Static	Present	Present	f = 100%	Not Applicable
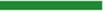	Positive	Absent	Present	0% < f ≤ 100%	≥5
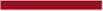	Weak negative	Present	Present	f ≥ 80%	Not Applicable
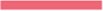	Negative	Present	Present	0% < f < 80%	Not Applicable
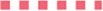	Strong negative	Present	Absent	f = 0%	Not Applicable
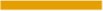	No N-mers data	Present	No data	No data	Not Applicable
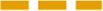	No 2-mers data	No data	Any	Any	Not Applicable
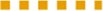	Indirect	Absent	Present	f > 0%	<5

Each detected PPI is assigned to a class only if it satisfies the criteria defined in the table. Style: assigned style for each PPI class, which is used in graphical representations. PPI Dynamics: assigned name for each PPI class. PPI in 2-mers: binary outcome of PPI prediction using 2-mers. PPI in N-mers: binary outcome of PPI prediction using N-mers. N-mers PPI Frequency: relative frequency (%) in which the PPI needs to be detected in N-mers. N° of contacts in N-mers: minimum number of contacts between the PPI pair. Note: The rationale for these categories is to distinguish meaningful values and patterns with visual clues. For example, a static interaction does not vary under any modelling circumstance, as it always appears if the protein pair is present in a prediction. A black, solid, thick line is assigned, highlighting the persistence or invariance of the PPI despite the introduction of additional proteins into the system. A strong negative is the opposite: it is present in the simple dimeric prediction, but consistently disappears in more complex combinations. A thin, dashed, red line is assigned for this type, symbolizing the extreme sensitivity of the PPI to the introduction of additional proteins into the system. For positive interactions, a simple thickness gradient that varies depending on frequency is set (More thickness = more frequency). For negative interactions, we initially considered applying the same thickness gradient, but for some pairs we observed that AlphaFold occasionally fails to predict the expected PPI in a subset of unstable N-mer combinations without any significant association to any partner (see supplementary material). Due to this, we decided to add a 20% frequency margin as a buffer to distinguish between two categories: 80 ≤ f < 100 for those that only a small proportion of predictions fail and 0 < f < 80 for those with a considerable fraction of predictions failing to recapitulate the 2-mer PPI mode between the protein pair.

Figure 8a shows an example of the resulting PPI graph for the system composed of the chaperone Hsp83/Hsp90 and cochaperones Aha1 and Sti1. On the one hand, Hsp90 is known to form a homodimeric subcomplex (Jamabo et al., 2022). This is captured by MultimerMapper and represented by a self-connecting edge with an inset that indicates that the homodimer is stable but the homotrimer isn’t. Also, if at least two subunits of Hsp90 are present, they are predicted to interact with each other no matter the context, as the edge is classified as static. On the other hand, both Aha1 and Sti1 are known to interact with the core Hsp90 dimer through multivalent interactions, forming the heterodimers Hsp90_2_:Aha1_2_ and Hsp90_2_:Sti1_2_ (Mondol et al., 2023; Prodromou et al., 1999), which is predicted by MultimerMapper and represented with the nomenclature 2P2Q. Notice that, for both interactions, one of the binding modes isn’t detected in the dimeric context, which is represented as a green edge. Also, Aha1 is predicted to be interacting with itself, but the homodimer and homotrimer are predicted to be unstable in isolation. The reason is that Aha1 can only interact with itself if Hsp90 is present in the predictions (see below).

**FIGURE 8:**
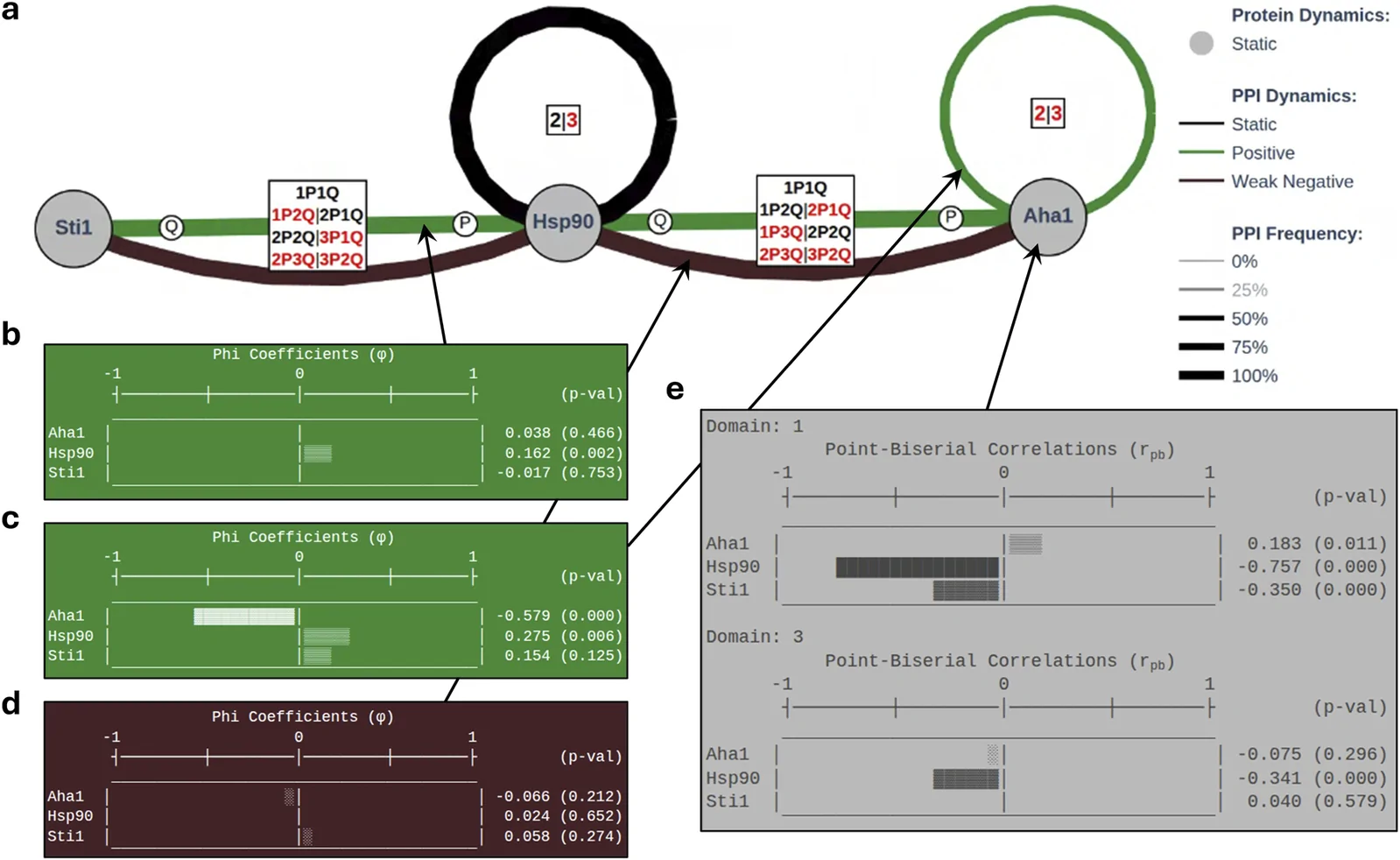
Interactive PPI graphs. PPI graph for the system formed by the Hsp83/Hsp90 chaperone (ID: Tb11.v5.0543) and co-chaperones Sti1 (Tb927.5.2940) and Aha1 (ID: Tb927.10.13710) from *T. brucei*. **(a)** Resulting PPI graph. Insets show the identified stability of homooligomeric and multivalent heterooligomeric subassemblies. Stable stoichiometries are black and unstable red. For multivalent pairs, circles labeled P and Q indicate the correspondence between the stoichiometric coefficients shown in the insets and the respective proteins. Bar plots showing the φ coefficients of the positive dynamic PPI between Sti1 and Hsp90 **(b)**, the positive dynamic homooligomerization of Aha1 **(c)** and the weak dynamic negative PPI between Aha1 and Hsp90 **(d)**. p-values are shown between brackets. **(e)** Bar plots with $r_{pb}$ coefficients for the structured domains of Aha1.

#### 3.8.2 Association between PPI dynamics and partners ( φ coefficient)

To analyze the statistical significance of dynamic PPIs, MultimerMapper conducts a relationship analysis that associates the presence or absence of protein partners with the presence or absence of PPIs. For this, a phi-coefficient ( φ ) and its statistical significance using the $\chi^{2}$ test is computed (Chedzoy, 2006). The φ coefficient is built in a way that values significantly higher than zero (a p-value is provided) for a particular partner means that the partner is positively associated with the PPI. In other words, its presence is associated with the engagement of that particular PPI, and a high φ value indicates that the correlation is strong. On the other hand, when this value is significantly smaller than zero, its presence is negatively associated with the engagement. This means that its presence is associated with the disruption of the PPI.

This data is included in the form of bar plots on each PPI mode metadata (Figures 8b–d). For the chaperone example, one of the PPI modes between Sti1 and Hsp90 is positively associated with Hsp90 presence (Figure 8b), as this PPI only appears when the models contain multiple subunits of Hsp90 (*i.e.*, the dimer). Something similar happens for the homooligomerization of Aha1, being positively associated with Hsp90 presence Figure 8c, as it only appears when there are two subunits of Hsp90. Notice that Aha1 is negatively associated with its homointeraction. This is because predictions that only contain Aha1 do not contain the PPI. Also, the φ value for Sti1 is positive, but its p-value isn’t significant. On the other hand, one of the PPI modes between Aha1 and Hsp90 is classified as weak dynamic negative, but no association is significant (Figure 8d).

#### 3.8.3 Association between domain conformation and partners ( $r_{\text{pb}}$ coefficient)

Besides associating protein partners with predicted PPI dynamics, MultimerMapper also correlates the presence of specific partners with variations in RMSD using the point biserial coefficient ( $r_{\text{pb}}$ ). This correlation is performed only for well-structured domains, excluding disordered loops from the RMSD calculations. Similarly to the φ coefficient: it varies between −1 and 1, with $r_{\text{pb}}$ values significantly bigger than zero indicating a correlation between increase in RMSD (with respect to the reference domain structure) and the partner. Absolute values closer to 1 indicate a strong correlation and negative values are interpreted in the opposite way. For example, Aha1 has two structured domains, and MultimerMapper detects that both suffer conformational variations that lowers their RMSD and are associated with the presence of Hsp90 (Figure 8e). This association reflect conformational changes induced in Aha1 upon binding to Hsp90.

Through the association of PPI patterns and conformational changes with specific partners, φ and $r_{\text{pb}}$ values aid in the identification of competitive binding, inhibition and activation of PPIs scenarios. Potentially, conformational changes can be induced by the implicated partners, giving the user an intuitive and systematic way to obtain bioinformatic evidence with statistical support, and develop functional hypothesis derived from the conformational sampling generated using this context-dependent structure prediction method.

## 4 Discussion

This work presents a novel way to systematically analyze predicted structures generated using deep learning structural tools. It relies on the statistical analysis of associated confidence metrics and atomic coordinates to predict interactions and context-dependent conformational changes. The methodology is based on the systematic exploration of the stoichiometric space using an algorithm that seeks to balance the use of computational resources while maximizing the possibility of finding variations in protein interaction patterns and conformational states. In turn, the algorithm deciphers which protein combinations produce complexes where all the predicted subunits interact with each other, classifying them as “stable”. By adding new subunits to these stable combinations, we can discern those pathways that produce unstable combinations and discard them, reducing the exploration space. Ultimately, it is possible to identify the terminal combinations of these branches, which in theory have no further possible interaction surfaces with any of the protein entities in the system. This gives rise to the combinations here mentioned as “convergent stoichiometries.” Furthermore, if the system contains multiple convergent stoichiometries, MultimerMapper is able to detect them.

To support these claims, we tested all three predictive layers of MultimerMapper against systems outside trypanosomatids (Supplementary Material). For stoichiometry inference, using 20 hetero-oligomeric complexes randomly sampled from the Complex Portal database across five species, the expected stoichiometry was recovered among the stable stoichiometries in 80% of cases, considering an average of 3.55 (median = 2) stable stoichiometries identified per system containing all queried entities, and could be unambiguously differentiated in 60% of cases. A stoichiometry was considered unambiguously differentiated if it was the only convergent stoichiometry returned by the SSE algorithm (75% of successful cases), or if it was a convergent stoichiometry among multiple convergent candidates and was uniquely resolved using confidence-profiles and/or symmetry-fallback analysis (25% of successful cases). For context-dependent conformational changes, MultimerMapper correctly predicted the literature-described rearrangement in 6 of 7 tested systems (Supplementary Videos S4–10), correctly associating the expected proteins with the expected conformations using the BPD, only failing in an inter-species case, consistent with the reduced coevolutionary signal available to AlphaFold for such pairs (Li et al., 2025). For PPI dynamics classification, the expected static/negative/positive behavior of the target PPIs was correctly recovered in 8 of 12 tested systems. The failures were attributable to general PPI false negatives, the presence of inter-species proteins and a genuine ternary-intermediate mechanism outside our binary static/dynamic framework. However, we saw a tendency to overpredict negative PPI dynamics (∼20%) due to drops in model confidence in unstable stoichiometric contexts that nonetheless retain structurally correct atomic coordinates for the interaction (see Supplementary Material). Together, these results indicate that MultimerMapper’s outputs generalize beyond the trypanosomatid systems used for development.

The fact that AlphaFold can add extra subunits beyond the expected point for symmetric architectures could result in stable stoichiometries that differ from reported stoichiometries. To overcome this, MultimerMapper implements other strategies useful to discern between potential stoichiometries. These are the symmetry fallback detection and the pairwise confidence metric profiles. Either way, MultimerMapper’s predictions can only be as good as the capabilities of the method used to generate the structures. While our measured PPI prediction sensitivity, specificity and area under curves are similar to that reported in other works (Bryant et al., 2022a; Schmid et al., 2025; Todor et al., 2026), there is still a considerable proportion of true interactions that escape detection, a gap that future advances in structure prediction accuracy is expected to narrow. Regarding size constraints, when AF3 server is used (Abramson et al., 2024), the size limit of the convergent stoichiometries that can be detected is approximately 5000 amino acids. Also, systems containing too many proteins would be computationally costly, as the number of predictions required is directly proportional to the square of the number of entities. Therefore, it’s advisable to use as an initialization system a set of protein entities with high interacting probability (*e.g.*, Co-IP). If complex size becomes a limiting factor, MultimerMapper can be used to define sub-stoichiometries, which can then be used in combination with CombFold to reconstruct full assemblies (Shor and Schneidman-Duhovny, 2024; Rodríguez Araya et al., 2026). Nonetheless, trends in deep learning structural models evolution indicate that they will become increasingly efficient and precise, making it possible to model larger systems more quickly and accurately.

MultimerMapper is also capable of constructing pseudo-trajectories based on the conformational states of individual proteins. While there is still debate as to whether conformational sampling using AlphaFold is truly capable of capturing conformational changes (Chakravarty et al., 2024), to our knowledge, our methodology is fundamentally different from what has been described so far (M et al., 2024; Wayment-Steele et al., 2024; Agarwal and McShan, 2024). Previous methodologies sample the input MSA by eliminating coevolutionary information, which is ultimately reflected in the loss of predicted contacts, increasing the conformational variability on individual monomers. In contrast, MultimerMapper generates conformation variation through contextual sampling of the MSA. That is, systematically varying the subunits present in the predictions, without explicitly eliminating intramolecular coevolutionary information. This new perspective is what allows to correlate the context with the conformational states and confidence metrics of proteins. These variations not only provide further evidence of PPI engagement but can be associated with protein functionality in the context of multiprotein complexes, being of great value for large, dynamic systems, where molecular dynamics is computationally costly, particularly when the objective is to analyze protein behavior in multiple contexts. In this sense, MultimerMapper systematize this at low computational cost.

Because the SSE algorithm was designed to reduce computational cost by exploring only stable branches, the total number of chains in the overall models is not equal for each protein entity. This has a clear implications for the interpretation of BPD values, since protein entities that are underexplored will tend to present negative values without actually being negatively associated. In this sense, BPD values close to −1 could only be validly associated with the absence of a protein if, at some point in the pseudo-trajectory, their values reach clearly elevated and sustained positive values. Therefore, it would be necessary to find an appropriate statistical framework to define the significance of BPD values in future studies. A related caveat applies to the φ and $r_{\text{pb}}$ statistics. In both cases, the unit of observation is the individual model rather than the prediction from which it derives. Since each prediction yields multiple models sharing the same MSA, it can be argued that models are not fully independent observations, which would make the associated p-values anti-conservative. For the φ coefficient, we provide a parameter (Table 1) to instead aggregate models per prediction before computing the contingency table, at the cost of substantially reducing the number of observations available for edges with sparse data; no equivalent aggregation is currently implemented for the point-biserial correlation, as no principled criterion exists for reducing multiple structurally distinct models of a given context to a single RMSD value. Given this, the φ and point-biserial statistics reported here should be interpreted as exploratory rather than strictly confirmatory, and are best used as a hypothesis-generating tool to prioritize partners for further investigation rather than as definitive statistical evidence of association.

Although the current methodology only considers polypeptide entities, it is feasible to extend it to other molecular entities. To do that, metrics analogous to the BPD could be generated, but measuring the bias of small molecules or ions to associate their presence or absence with the conformational state of different molecular entities. For example, by predicting the structure of guanine quadruplex forming DNA sequences using different numbers and types of ions (Nishio et al., 2021), we have observed how these ions are associated with different quadruplex conformations (data not shown).

With respect to identifying multiple binding modes between protein pairs, MultimerMapper conducts the clustering algorithm only if it has predicted multivalency. If a pair is considered monovalent, the clustering is not conducted, and all available contact matrixes are considered as a single cluster. In fact, multiple binding modes are not always linked to multivalency. For example, some proteins can interact with the same surface via multiple conformational states (Barrera-Vilar et al., 2022). The main difficulty for these cases is to distinguish between a single cluster versus multiple ones. One possible solution would be performing the hierarchical clustering using a cluster size of 2 and applying an RMSD threshold between the resulting representative structures. If these two potential binding modes differ significantly, i.e., they surpass the RMSD threshold, the default matrix clustering algorithm can be triggered to identify the number of different binding modes. If not, they would be considered as a single binding mode. This solution would require to find a suitable RMSD cutoff using a reliable benchmark dataset and is something that we’re considering for inclusion in future versions of the software.

This program was designed to assist in the systematical interpretation of structural predictions, particularly designed for users without extensive experience in structural and computational biology. As such, the independence from third-party software was intended for ease of use. Therefore, each run generates an interactive HTML-based graphical report that can be executed in any browser. It has integrated structure viewers and interactive graphs that allow for easy interpretation of the behavior of the system of interest. For advanced users, the software can also be incorporated into other bioinformatics pipelines through the various packages available in MultimerMapper. In addition, all raw output data are saved to an organized filesystem, enabling independent downstream analyses.

In summary, this tool implements a novel methodology that can be well adapted by the scientific community to understand the behavior of molecular systems involving the simultaneous interaction of multiple proteins. It does not require computer expertise or sophisticated structural tools, being particularly useful for analyzing completely unknown and highly dynamic systems, as it requires no prior information beyond their amino acid sequences. In future iterations, it will be possible to integrate other types of molecules of biological interest and evaluate their association with the conformational behavior of different biopolymers.

## Data Availability

The source code of MultimerMapper can be found at https://github.com/elviorodriguez/MultimerMapper and the final stable version used in this work was uploaded to Zenodo (https://doi.org/10.5281/zenodo.22031831). The code used to generate the sequence alignments and protein structure predictions with AlphaFold2-multimer and the modified Discoba database can be found at https://github.com/elviorodriguez/DiscobaMultimer. The code to generate the negative dataset for PPI prediction can be found at https://github.com/elviorodriguez/negative_PPI_pairs_generator. The code to sample complexes from the Complex Portal database is available at https://github.com/elviorodriguez/random_complexes_for_benchmark.
