# Supplementary material for: Context-dependent protein structure prediction analysis and complex stoichiometry inference with MultimerMapper
Source: Front Bioinform. 2026 Oct 7;6:1914731. doi: 10.3389/fbinf.2026.1914731 (PMC13688643; doi:10.3389/fbinf.2026.1914731)
Supplement: Supplementary file 3 [file DataSheet3.zip › Supplementary_file_3-MRGBP_trajectory.html]

MRGBP Combined View
